# Ferrets exclusively synthesize Neu5Ac and express naturally humanized influenza A virus receptors

**DOI:** 10.1038/ncomms6750

**Published:** 2014-12-17

**Authors:** Preston S.K. Ng, Raphael Böhm, Lauren E. Hartley-Tassell, Jason A. Steen, Hui Wang, Samuel W. Lukowski, Paula L. Hawthorne, Ann E.O. Trezise, Peter J. Coloe, Sean M. Grimmond, Thomas Haselhorst, Mark von Itzstein, Adrienne W. Paton, James C. Paton, Michael P. Jennings

**Affiliations:** 1 Institute For Glycomics, Griffith University, Gold Coast, Queensland 4222, Australia; 2 Australian Infectious Diseases Research Centre, The University Of Queensland, Building 76, St Lucia, Queensland 4067, Australia; 3 Research Centre for Infectious Diseases, School of Molecular and Biomedical Science, Molecular Life Sciences Building, North Terrace, University of Adelaide, South Australia 5005, Australia; 4 University Of Geneva, Faculty of Medicine, Geneva University Medical Centre, 1, rue Michel-Servet, CH-1211 Geneva 4, Switzerland; 5 Australian Equine Genetics Research Centre, The University of Queensland, Building 68, Level 7, St. Lucia, Queensland 4072, Australia; 6 School Of Science, Engineering and Health, RMIT University, Building 14, 124 La Trobe Street, Melbourne, Victoria 3000, Australia; 7 Queensland Centre for Medical Genomics, Institute for Molecular Bioscience, The University of Queensland, St Lucia, Brisbane, Queensland 4072, Australia; 8 Wolfson Wohl Cancer Research Centre, Institute for Cancer Sciences, University of Glasgow, Garscube Estate, Switchback Road, Bearsden, Glasgow G61 1BD, UK

## Abstract

Mammals express the sialic acids *N*-acetylneuraminic acid (Neu5Ac) and *N-*glycolylneuraminic acid (Neu5Gc) on cell surfaces, where they act as receptors for pathogens, including influenza A virus (IAV). Neu5Gc is synthesized from Neu5Ac by the enzyme cytidine monophosphate-*N*-acetylneuraminic acid hydroxylase (CMAH). In humans, this enzyme is inactive and only Neu5Ac is produced. Ferrets are susceptible to human-adapted IAV strains and have been the dominant animal model for IAV studies. Here we show that ferrets, like humans, do not synthesize Neu5Gc. Genomic analysis reveals an ancient, nine-exon deletion in the ferret *CMAH* gene that is shared by the Pinnipedia and Musteloidia members of the Carnivora. Interactions between two human strains of IAV with the sialyllactose receptor (sialic acid—α2,6Gal) confirm that the type of terminal sialic acid contributes significantly to IAV receptor specificity. Our results indicate that exclusive expression of Neu5Ac contributes to the susceptibility of ferrets to human-adapted IAV strains.

## Abstract (web summary)

Ferrets constitute a useful model for influenza research because they are susceptible to human-adapted flu viruses. Here, the authors show that ferrets, like humans, lack a functional CMAH enzyme and synthesize a single type of sialic acid (Neu5Ac), resulting in naturally humanized influenza virus receptors.

## Graphical abstract

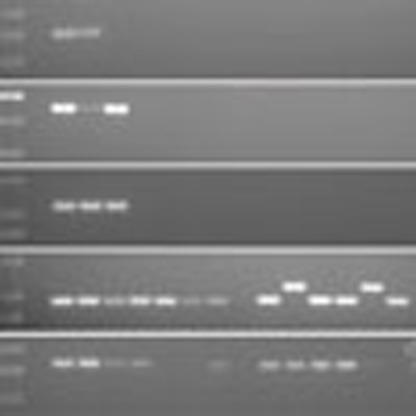

Influenza A virus (IAV) remains the most serious infectious disease threat to human health. Seasonal IAV kills 250,000–500,000 people each year worldwide. However it is the potential for the emergence of highly virulent pandemic IAV strains, such as the 1918A/H1N1 strain that killed 20–40 million people^1^, which illustrates the grave risk posed by this pathogen. IAV is a member of the family Orthomyxoviridae and has a negative-sense, single-stranded and segmented RNA genome. IAV antigenic diversity is high, with mutations accumulating during viral replication (antigenic drift) and by exchange of genomic material between IAVs co-infecting the same cell (antigenic shift). Therefore IAVs are further subtyped based on antigenic differences in the two membrane glycoproteins: haemagglutinin (HA) and neuraminidase (NA). HA is responsible for the initial attachment of the virus to the host cell membrane by binding to sialic-acid (SA) receptors, while NA ensures mobility of virus in the respiratory tract and release of new viral progeny by its sialic-acid cleavage activity^2^. Sequence variations in these proteins may alter IAV host range and virulence by changing their specificity for the spectrum of distinct HA^3^ receptor structures and NA substrates^4^ on the cells, tissues and organs of vertebrate hosts. This continual and rapid IAV evolution results in the emergence of new strains from animal reservoirs to infect humans; the lack of protective immunity from previous IAV infections; the requirement for regular reformulation of IAV vaccines; and the generation of IAV resistance to anti-viral drugs^5^.

Detailed study of human IAV was not possible until 1933 when it was first isolated by infection of ferrets (*Mustela putorius furo*) with nasal washings from human IAV patients^6^. Over the past 80 years ferrets have remained the dominant model system^7^ for study of IAV due to their unique susceptibility to human IAV strains^8^. The majority of studies of IAV host species adaptation has focused on the type of linkage between the sialic acid and the penultimate galactose residue, SAα2,6Gal or SAα2,3Gal, and the distribution of these receptors in the host^9^,^10^. The susceptibility of ferrets for human IAV strains has been ascribed to its similar distribution of SAα2,6-linkage receptors in the respiratory tract^11^. The ferret continues to serve as the key animal model system for IAV, including for a series of recent, high-profile transmission studies^12^,^13^. A complete understanding of ferret susceptibility to IAV is therefore essential for research on this significant human pathogen.

Neu5Gc is present in most vertebrates^14^,^15^, but is absent in humans^16^,^17^ due to a mutation that inactivates cytidine monophosphate-*N*-acetylneuraminic acid hydroxylase (CMAH)^18^, the enzyme that converts cytidine monophosphate-*N*-acetylneuraminic acid (CMP-Neu5Ac) to Neu5Gc^19^. There is a growing recognition that the type of terminal sialic acid also plays a role in IAV–receptor interaction^10^,^20^.

Here we show that ferrets, like humans, lack an active CMAH and do not produce Neu5Gc. We show that most of the ferret *CMAH* gene has been deleted by an ancient mutation shared by several other members of the order carnivora. Analyses of whole human IAV with fully characterized IAV receptor structures confirm the importance of Neu5Ac in both HA and NA functions, and that exclusive expression of Neu5Ac is a contributing factor to the unique suitability of ferrets as a model for human-adapted IAV.

## Results

### Ferrets do not express Neu5Gc

**Figure 1:**
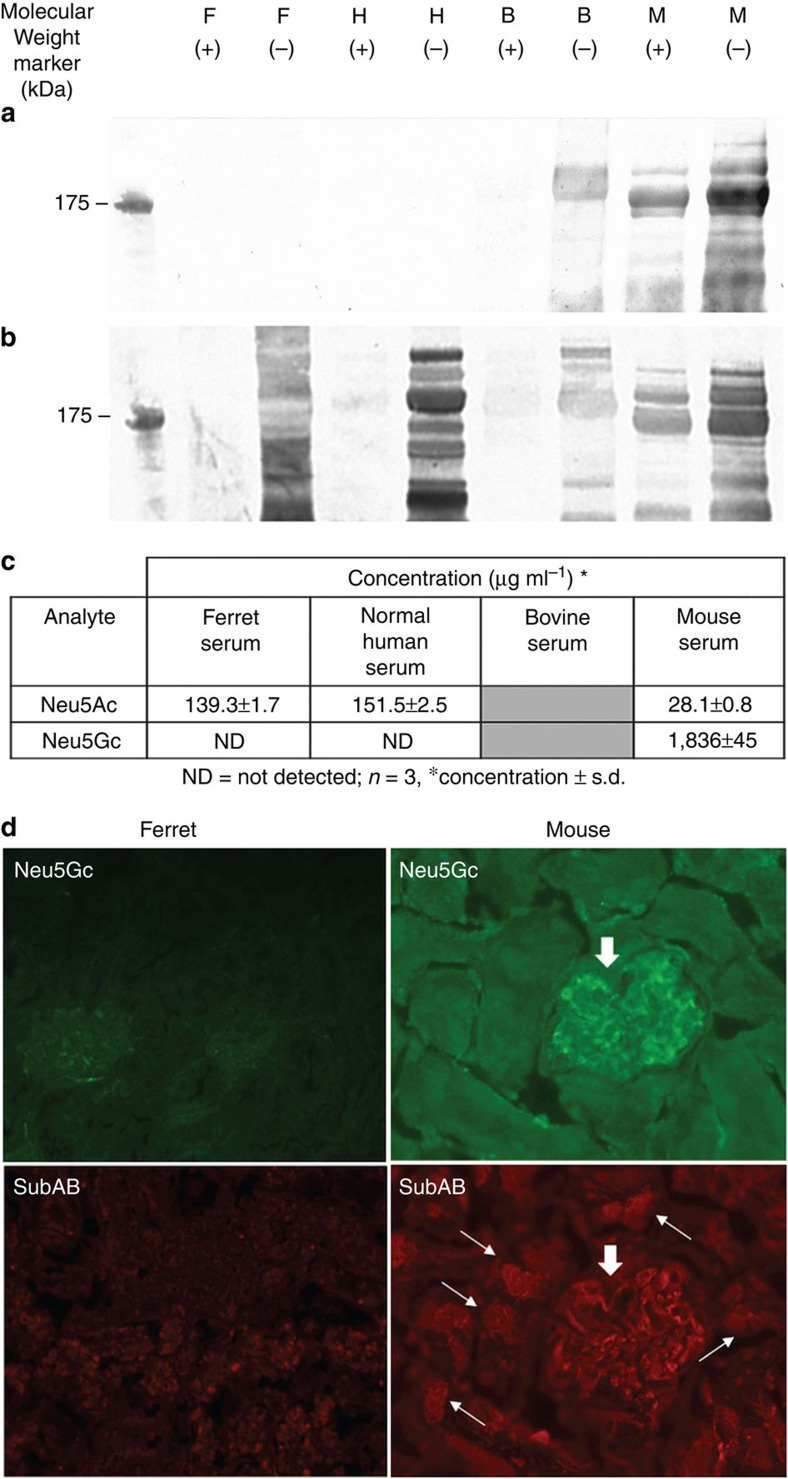
Analysis of sialic acid in ferret and other mammalian species. (**a**) Western blot analysis showing the absence/presence of Neu5Gc in serum samples (10 μg) when tested with anti-Neu5Gc antibody. (**b**) Western blot analysis of serum samples when tested with sialic-acid-specific lectin, SNA (**c**) Sugar analysis showing amount of Neu5Ac and Neu5Gc in serum samples. (**d**) Double immunostaining of Neu5Gc in mouse and ferret kidney tissue using chicken anti-Neu5Gc (Neu5Gc panels) and SubAB overlay followed by rabbit anti-SubA (SubAB panels). In **a** and **b**, F=ferret, H=human, B=bovine and M=mouse serum, respectively; + or − represent samples with or without neuraminidase treatment (see Supplementary Fig. 1 for full western blot images).

We developed the hypothesis that a contributing factor to the susceptibility of ferrets to human strains of IAV may be the type of sialic acid they express. To explore this hypothesis, initial studies were conducted using serum samples from ferret and other species known to express either Neu5Gc or Neu5Ac^14^. Western blot with Neu5Gc-specific immunoglobulin (Ig)Y antibody revealed reactivity in murine and bovine serum, but not human or ferret samples (Fig. 1a). Western blots of samples from these species were probed with *Sambucus nigra (SNA)*, a lectin that does not discriminate between these two types of sialic acid (Fig. 1b), demonstrating that sialic-acid residues are present on serum glycoproteins in all species. The same serum samples were analysed by high-performance liquid chromatography to quantitate the amount and type of sialic acid present. Both ferret and human serum had Neu5Ac, but no detectable Neu5Gc, while mouse serum contained predominantly Neu5Gc (Fig. 1c). Further investigation was conducted using cryopreserved tissue sections from brain, lung, kidney, liver and spleen prepared from healthy ferrets and mouse. These sections were probed with Neu5Gc-specific IgY antibody, and also with SubAB, an AB5 toxin with a B subunit that selectively binds Neu5Gc carbohydrate structures^10^,^21^. These studies revealed abundant staining of Neu5Gc in all mouse tissues using both methods (Fig. 1d; Supplementary Fig. 2a–g), but no staining was observed in the equivalent ferret tissue samples (Fig. 1d; Supplementary Fig. 2h,i). Taken together, these data show that, like humans, ferrets do not express Neu5Gc.

### The ferret *CMAH* gene is deleted

**Figure 2:**
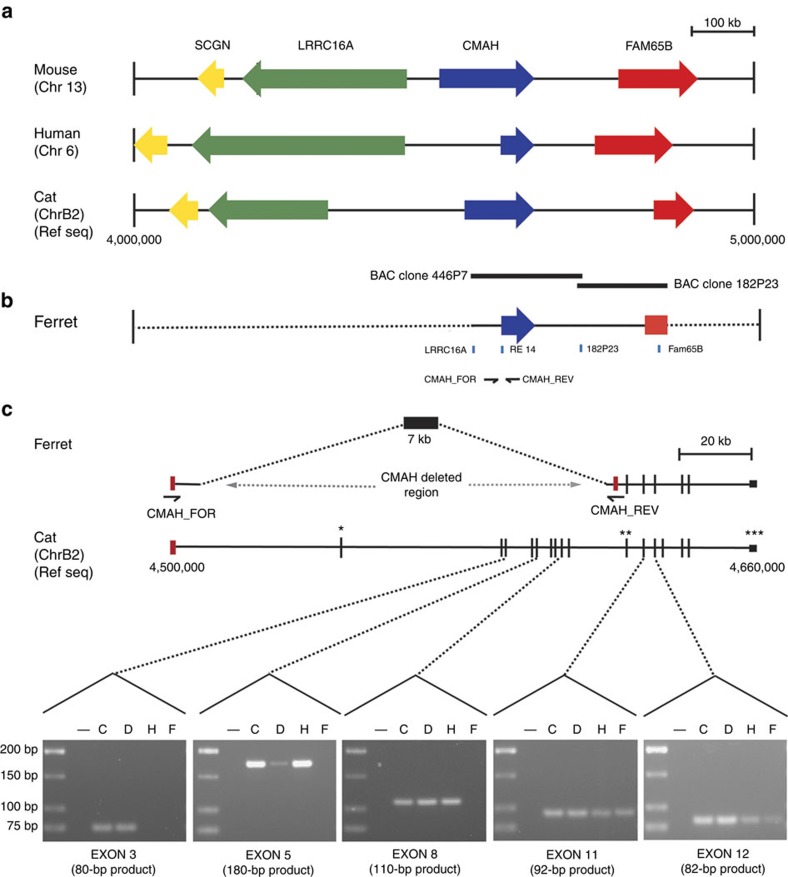
Genomic analysis of a deleted region in the ferret*CMAH* gene responsible for the absence of Neu5Gc in both ferrets and humans. (**a**) Synteny in the *CMAH* region of mouse, human and cat genomes. (**b**) Representation of the *CMAH* region of the ferret genome characterized in this study. Black bars represent region spanned by BAC clones 446P7 and 182P23. Blue bars indicate location of probes used in screening BAC library. Arrows indicate position of primers used to amplify the PCR product spanning the *CMAH*-deletion region. (**c**) Comparison of the *CMAH* region in cat and ferret genomes. Seven-kb PCR product containing the *CMAH*-deleted region was amplified using primers CMAH_FOR and CMAH_REV, respectively. (*) indicates the start of the *CMAH* gene, (**) indicates individual exon(s) on the *CMAH* gene and (***) indicates the end of the *CMAH* gene. Images below show *CMAH* exon PCR products from exon 3, exon 5, exon 8, exon 11 and exon 12, from cat (C), dog (D), human (H) and ferret (F) genomic DNA.

To determine the molecular basis for the lack of Neu5Gc expression in a ferret, we investigated the ferret *CMAH* gene. Synteny in the *CMAH* region is well conserved in mammalian genomes, with the same genes present in the *CMAH* flanking regions of eukaryotes (Fig. 2a) and the ferret (Fig. 2b). The coding sequence of *CMAH* is also well conserved. Primer sets to amplify exons from all mammalian *CMAH* genes were designed based on the most conserved exons (exons 3, 5, 8, 11 and 12; Fig. 2c). All of the exons amplified from the carnivore species cat and dog genomic DNA. All except exon 3 amplified from human genomic DNA. This region corresponds to the deletion event that inactivated the human *CMAH* gene resulting in the loss of Neu5Gc biosynthesis^18^. Only exons 11 and 12 amplified from ferret DNA, suggesting that there may be a large deletion in ferret *CMAH*. A ferret bacterial artificial chromosome (BAC) clone library was screened using probes specific for conserved regions flanking *CMAH* in related carnivore genomes (Fig. 2b), resulting in the isolation of the BAC clone 182P23. Sequence analysis of this clone facilitated design of a probe that was used to isolate BAC clone 446P7. These two BAC clones were sequenced using single-molecule real-time (SMRT) sequencing technology, resulting in two complete sequences that overlapped and covered the entire *CMAH* region. Sequence analysis identified a large deletion that results in loss of the first nine coding-sequence exons of *CMAH* in the ferret genome, and multiple stop mutations in exon 11. The deletion is consistent with the exon PCR amplification data (Fig. 2c). Primers were designed at the boundaries of the deleted region and used in PCR to confirm that the deletion exists in independent individual ferrets. Recent data available from the Broad ferret genome project (http://www.broadinstitute.org/annotation/genome/ferret, accessed 9 July 2014) are consistent with data presented here, but do not currently annotate the *CMAH* deletion. We conclude that the lack of Neu5Gc expression in ferrets is due to deletion of the majority of the *CMAH* gene.

### The ferret *CMAH* deletion is an ancient mutation

To determine the evolutionary origin of the *CMAH*-deletion event in ferret, we used the same *CMAH* exon primer set to examine genomic DNA from 15 Mustelidae species selected to cover all genera. All showed the same profile as ferret (Supplementary Fig. 3a–c), suggesting that the *CMAH*-deletion event occurred prior to the divergence of the Pinnipedia and Musteloidea lineages. The analysis was then widened to include the other members of the Arctoid lineages, Ursidae and Pinnipedia^22^, and revealed that all members of the Pinnipedia tested also shared the same *CMAH* deletion as the Musteloids (Supplementary Fig. 3b). The Ursidae species *Ursus americanus* (American black bear) contained all *CMAH* exons tested as did *Urocyon cinereoargenteus* (grey fox), which is the basal species of the Canidae family^23^ (Supplementary Fig. 3a,b). We propose that the *CMAH* mutation occurred in the infraorder Arctoidea after divergence of the Ursidea from the Pinnipedia and Musteloidea lineages, dating the mutation to between 38 and 40 million years ago^24^. Our data are consistent with and support studies that propose that the Ursidea descended from an Arctoidea ancestor separate from Pinnipedia and Musteloidea^24^,^25^. The absence of Neu5Gc expression has also been observed in chickens^26^, reptiles^14^, various species of birds^14^ (with the exception of ducks^27^), the platypus^14^, in western dog breeds^28^,^29^ and recently in new world monkeys^30^. An inactive *CMAH* allele results in loss of CMAH expression that generates blood group antigen differences in cats^31^,^32^. It has been proposed that the loss of Neu5Gc expression in humans may have resulted from selective pressure from pathogens that utilize sialic-acid-containing receptors^33^,^34^,^35^. Our finding that two major families of carnivores also lack Neu5Gc expression, and that this event preceded the human *CMAH* mutation, which occurred only ~3 million years ago^34^, suggests that this selective pressure has been present throughout evolutionary history of vertebrates. This finding is also consistent with the hypothesis that inactivation of CMAH is a crucial speciation event, as this mutation may generate reproductive incompatibility^36^.

### Terminal sialic-acid type and linkage affect IAV interactions

To a large extent, IAV host range has been seen through the prism of IAV receptor type and distribution in the host. The location of these receptors in host organs and tissues dictate the type of pathology. Sialic-acid receptor recognition by both HA and NA play an important role in maintaining a balance for successful infection^37^,^38^. In addition to the linkage type, that is SAα2,3Gal or SAα2,6Gal, the type of sialic acid has also been suggested to have an influence in IAV host range^39^. This is supported by erythrocyte agglutination assays using red blood cells expressing distinct sialic-acid types^40^,^41^,^42^, IAV-binding assays with Neu5Ac or NeuGc receptors^43^,^44^,^45^, determination of sialic-acid cleavage rates with Neu5Ac or NeuGc receptors^46^, amino-acid modifications of HA^47^ and NA^4^. A fundamental difference between non-Neu5Gc-expressing IAV hosts, such as human and ferret, and the Neu5Gc-expressing IAV reservoirs, such as pig and duck, is that the latter two species express both Neu5Gc- and Neu5Ac-terminated receptors. Selective pressure for adaptation in humans is therefore restricted to Neu5Ac receptor, however, in pigs and ducks IAV can adapt to either Neu5Ac and Neu5Gc receptors.

**Figure 3:**
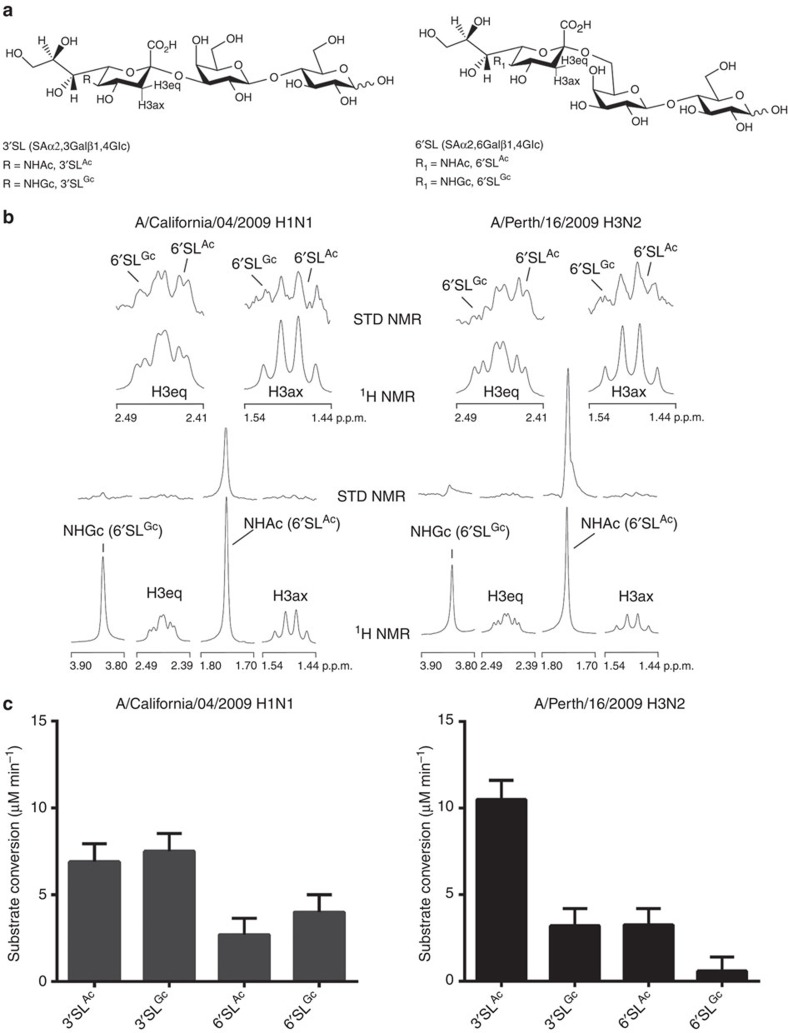
Molecular analysis demonstrating the importance of sialic-acid (SA) type on HA and NA specificity. (**a**) Chemical structures of SAα2,3Gal and SAα2,6Gal in which SA is either Neu5Ac (3′SL^Ac^, 6′SL^Ac^) or Neu5Gc (3′SL^Gc^, 6′SL^G^c). (**b**) Haemagglutinin receptor specificity of human influenza A viruses pH1N1 and H3N2. ^1^H NMR (bottom row) and STD NMR (above) spectra were obtained of an equimolar mixture of 2 mM 6′SL^Ac^ (Neu5Acα2,6Galβ1,4Glc) and 6′SL^Gc^ (Neu5Gcα2,6Galβ1,4Glc) with pH1N1 virus (A/California/04/2009, left panel) and H3N2 virus (A/Perth/16/2009, right panel), respectively. All NMR samples also contained a low concentration of oseltamivir carboxylate (50 μM, OC), a very potent nanomolar inhibitor of the viral neuraminidase to inhibit sialic-acid cleavage (Supplementary Fig. 4). Shown are only the axial and equatorial H3 protons (H3ax, H3eq) and the *N*-acetamido methyl (NHAc) and methylene (NHGc) protons of the sialic-acid moiety that are clearly distinguishable between 6′SL^Ac^ and 6′SL^Gc^. The entire spectra are shown in Supplementary Fig. 5. (**c**) Neuraminidase substrate specificity of human influenza A viruses pH1N1 and H3N2. ^1^H NMR spectroscopy was employed to follow the cleavage of sialosides (6′SL^Ac^; 6′SL^Gc^; 3′SL^Ac^; 3′SL^Gc.^) upon addition of pH1N1 virus (A/California/04/2009, left panel) and H3N2 virus (A/Perth/16/2009, right panel), respectively. The conversion rate was calculated using the absolute peak intensity of the sialyllactose H3eq-signals (±7.5% error) based on substrate depletion by a successive series of ^1^H NMR spectra over 20 min at 37 °C (Supplementary Fig. 9).

To determine receptor-binding preference of human-adapted whole IAV and to resolve the relative roles of sialic-acid linkage type and sialic-acid species, we have conducted a series of saturation transfer difference (STD) nuclear magnetic resonance (NMR) experiments^48^. STD NMR is a versatile technique to investigate protein–ligand binding by saturating the protein resonances without effecting ligand signals. In the study presented here, STD NMR experiments were performed with intact influenza virus particles of two recently isolated human viruses (A/Perth/16/2009 H3N2 and A/California/04/2009 pH1N1), complexed with a mixture of 6′-sialyllactose (6′SL) synthetic ligands terminating in Neu5Ac (6′SL^Ac^) and Neu5Gc (6′SL^Gc^) (Fig. 3a,b). We and others have previously reported about using STD NMR to investigate ligand binding to intact virions^49^,^50^,^51^ and virus-like particles^52^,^53^,^54^. The results presented in this study are the first using whole intact influenza virus particles. In the current study, the interaction between HA displayed on whole IAV particles and sialyllactose ligands were analysed in the presence of a low concentration of oseltamivir carboxylate (OC; 50μM) to completely block the NA active site to prevent sialyllactose ligand binding to the NA and consequently sialic-acid cleavage^55^ (Fig. 3b, Supplementary Fig. 4).

Figure 3b shows ^1^H NMR and STD NMR spectra of an equimolar mixture of 6′SL^Ac^ and 6′SL^Gc^ in complex with whole IAV. Strong STD NMR signals for the 6′SL^Ac^-specific protons are observed, especially for the methyl protons of the *N*-acetamido group, for both H3 and 09H1 viruses. In contrast, 6′SL^Gc^ shows only very weak interactions with both viruses under identical experimental conditions. For both virus strains (pH1N1 virus (A/California/04/2009, left panel) and H3N2 virus (A/Perth/16/2009, right panel)) strong STD NMR signals are observed for the protons of the *N*-acetamido group (NHAc) (6′SL^Ac^), while the STD NMR signal intensities of the methylene protons (NGGc, 6′SL^Gc^) were generally very weak. Similarly, the STD NMR signal intensities of the H3eq and H3ax protons are stronger for 6′SL^Ac^ compared with 6′SL^Gc^ (magnified at the top). The results clearly demonstrate that pH1N1 and H3N2 IAVs show a strong preference for Neu5Ac-containing sialosides. To confirm that 6′SL^Gc^ has a very weak affinity to HA, we also conducted STD NMR experiments of 6′SL^Gc^ in the absence of 6′SL^Ac^ under otherwise identical experimental conditions (Supplementary Fig. 5). STD NMR control experiments of heat-treated virus and of OC in the absence of sialosides were performed to affirm that all observed STD NMR signals describe specific HA binding (Supplementary Fig. 6). Our NMR data with pure, fully characterized synthetic receptor structures confirm a profound preference for 6′SL terminating in Neu5Ac for two currently circulating human strains, consistent with adaptation of human IAV strains to Neu5Ac-terminated receptors. Data mining of glycan array results from the Consortium for Functional Glycomics (CFG) database (http://www.functionalglycomics.org) also revealed preferential binding by human IAV HA to Neu5Ac compared with Neu5Gc-terminating structures. Comparison of the binding of whole human-adapted IAV strains or purified HA to glycan arrays, displaying sialyllactose or sialyllactosamine structures, with identical spacer structures, terminating in either Neu5Ac or Neu5Gc also indicate a strong preference for Neu5Ac-terminating structures (Supplementary Figs 7 and 8).

The cleavage of four distinct sialyllactose substrates by whole influenza virus-associated NA was followed by a time-cause ^1^H NMR study. Figure 3c shows substrate conversion of 3′SL^Ac^, 3′SL^Gc^, 6′SL^Ac^ and 6′SL^Gc^ using identical virus preparations as used in the STD NMR experiments (Supplementary Fig. 9). Both viruses cleave α2,3-sialosides more efficiently than α2,6-sialosides. In case of the pH1N1 virus, the *N*-glycolyl substrates get converted slightly faster than the *N*-acetyl counterparts of the same linkage. On the contrary, *N*-acetyl-containing substrates are significant better substrates for the NA of the H3N2 virus compared with the *N*-glycolyl-containing substrates. Both linkage type and sialic-acid species contribute significantly towards NA specificity. For the H3N2 strain, the Neu5Ac-containing 3′SL^Ac^ and 6′SL^Ac^ are preferred over the equivalent substrate with Neu5Gc regardless of the linkage.

## Discussion

Our study reveals that ferrets are a naturally humanized model system with respect to IAV receptor biology. Previous studies have shown that ferrets have similar IAV receptors with respect to the SAα2,6-linkage^56^ and anatomical distribution^11^. Here we show that ferrets, like humans, exclusively express Neu5Ac on these receptors. Our STD NMR analysis of whole human IAV with fully characterized IAV receptor structures, and NA activity assays, confirm the importance of Neu5Ac in both HA and NA functions. Sub-optimal interactions of human-adapted IAV with Neu5Gc-terminated viral receptors, may explain why other dominant rodent animal models (mouse, rabbit, rat and guinea pig) are not optimal for studies with human-adapted IAV strains. We conclude that exclusive expression of Neu5Ac in ferrets is a contributing factor to their unique suitability as a model for human-adapted IAV. Recently, a CMAH mutation has been reported in new world monkeys, and this mutation differentiates them from old world monkey species, which express Neu5Gc^30^. This new finding supports the importance of exclusive expression of Neu5Ac-terminated receptors in human-adapted IAV model systems, as marmosets, a new world monkey species, have previously been shown to be suitable to study human-adapted IAV, including transmission studies, whereas, macaques, an old world monkey species, cannot transmit human-adapted IAV^57^. The implications of our discovery of the exclusive expression of Neu5Ac in ferrets extend beyond the IAV field. Ferrets may serve as a natural model system for other human pathogens that utilize sialic-acid receptors such as rotavirus^58^,^59^, and for studies on the emerging role of the Neu5Gc xeno-auto-antigen^60^ in inflammatory^61^, autoimmune^62^,^63^ and neoplastic human disease^64^.

## Methods

### Western blot analysis of sialic-acid expression in serum

Serum samples were purchased from commercial suppliers: ferret (Jomar Bioscience), human (H4522, Sigma), bovine (B8655, Sigma) and mouse (M5905, Sigma). Serum samples were diluted 1:10 in a 50-μl volume of 1 × NA buffer (N3786, Sigma), ±1 milliunit (mIU) NA (N3786, Sigma) and incubated at 37 °C for 3 h before SDS-polyacrylamide gel electrophoresis of 10 μg of each sample (NuPage 4–12% Bis-Tris gel, Invitrogen). For detection of Neu5Gc, primary antibody (1/2,000 dilution) and blocking solution (0.5% v/v in PBS) used was supplied by Sialix (formerly GC-Free Inc., San Diego, CA, USA). Secondary antibody used was anti-chicken IgY (IgG) alkaline phosphatase conjugate produced in rabbit (A9171, Sigma) at 1/10,000 dilution. For detection of sialic acid (Neu5Ac and Neu5Gc), lectin SNA-alkaline phosphatase conjugate (LA-6802-1, EY Laboratories) was used at 1/1,000 dilution in 1% bovine serum albumin (w/v) in PBS. All membranes were washed in 1 × tris-buffered saline, 0.05% Tween 20. Detection of bands with anti-Neu5Gc-specific sera or SNA that were present in NA (−) sample and absent in the NA (+) are interpreted as binding to serum proteins, with glycosylations terminated with sialic acid.

### Sugar analysis of serum for the detection of sialic acids

A 20-μl subsample of the stock samples was subjected to mild acidic conditions using 0.1 M trifluoroacetic acid. At the end of the reaction, the sample volume was reduced to dryness under vacuum and the residue was reconstituted in MilliQ water (100 μl). The analysis was carried out using a high-performance anion-exchange chromatograph with pulsed amperometric detection (HPAEC-PAD) fitted with a PA1 guard column (4 × 50 mm) connected to a CarboPac PA1 column (4 × 250 mm) held at 30 °C (ref. 65). The sample (10 μl) was injected into the HPAEC-PAD and analysed using a basic solvent (NaOH), at a flow rate of 1 ml min^−1^. The analytes detected were quantified using external calibration^66^. Samples were analysed in triplicate and the data averaged.

### Immunofluorescence labelling and microscopy

To generate cryopreserved tissue sections, organs including brain, lung, kidneys, liver and spleen were removed from a healthy mouse (balb/c male; 6 weeks old) or ferret (male; 2 years old) deeply anaesthetized by inhalation of Halothane (NRA Approval No, 40398/0198, Veterinary Companies of Australia PTY LTD). Experiments were approved by the Animal Ethics Committees of the University of Adelaide and the Institute of Medical and Veterinary Science, Adelaide, SA, Australia. The freshly removed organs were embedded in OCT Tissue-Tek (Sakura, USA), snap frozen in isopentane (APS Finechem, Australia) cooled by dry ice. Serial 7-μm sections were cut in an electronic cryotome (Thermo Electron Corporation, UK/USA). Mouse blood smears were made from fresh mouse blood. Both tissue sections and mouse blood smears were air dried, and stored in the airtight containers at −20°C before staining.

Cryopreserved sections or blood smears were fixed in 4% paraformaldehyde (Sigma, P6148) and permeabilized by 0.1% Triton X-100. The sections and blood smears were then incubated with 1 μg ml^−1^ SubAB (or PBS as a control) followed by BA nonspecific blinding blocking agent (GC-free Inc), primary antibodies (chicken anti-Neu5Gc or control serum, and rabbit anti-SubA) and secondary antibodies (goat anti-chicken-alexa 488 and goat anti-rabbit-alexa 594). All the incubations were carried out in a humidified atmosphere in the dark at room temperature. The tissue sections and blood smears were then examined with fluorescence microscope (Olympus AX 70 or Olympus IX-70) and the digital images were taken using the Precision Digital Imaging System (V++, Digital Optics Limited, Auckland, New Zealand) or Metamorph software program (version 6.3r7; Molecular Devices).

### Isolation of ferret *CMAH* region BAC clones

Comparison of regions shown in Fig. 2a was performed using genome sequences for mouse (accession code NC_000079.6), human (NC_000006.12) and cat (NC_018727.1), respectively. Outer probes indicated on Fig. 1b (sequences numbered as in GenBank accession codes KJ027518 and KJ027519) were designed in conserved regions flanking *CMAH* that were 100% homologous to other vertebrate species. These regions were amplified with primers (Supplementary Table 1) using ferret DNA (Zyagen GF-180, CA) as template using Go-Taq polymerase (Promega, M8291). PCR product was purified (Qiagen PCR Purification Kit, 28106) and sequenced using BigDye v3.1 (AB Biosystems). To label the probes, digoxigenin (DIG)-labelled versions of the respective forward primer (FOR) used in conjunction with a non-labelled reverse (REV) primer for each probe were used to amplify the DIG-labelled PCR product that was used as a probe for screening of a ferret BAC library (CHORI 237 BAC) ordered from the Children’s Hospital Oakland Research Institute (CHORI). PCR product was pooled, purified and quantitated prior to being used as a probe. The ferret BAC library was screened using 3 μg of DNA probe. Membrane prehybridization and hybridization steps were done according to Sambrook (Cold Spring Harbour, USA)^67^. Development of membrane was done using the Roche DIG Kit as per the manufacturer’s instructions (Cat no. 11745832910).

BAC DNA was extracted from positive clones using DNA PhasePrep BAC DNA Kit (Sigma, NA 0100). The BAC library was made in vector pBACGK1.1 and end sequenced with T7 and SP6 universal primers and Sanger sequencing using BigDye v3.1 (Applied Biosystems, 4336917). Selected clones were also sequenced by IonTorrent. One μg BAC DNA was fragmented to ~200–300 bp using a Covaris S2 ultrasonicator. Fragment libraries suitable for sequencing on the IonTorrent PGM were generated using the IonXpress Fragment library kit as per the manufacturer’s instructions (Life Technologies, Beverley, USA), and a single 314-chip of sequencing data generated for each BAC. Raw sequencing reads were trimmed for quality, reads mapping to either *E. coli* Dh10B or the BACX backbone were removed and a *de novo* assembly was generated using the CLC genomics workbench (CLC bio, Denmark). *De novo* contigs were mapped and visualized against the domestic cat (*Felis catus*) genome using BLAT and the UCSC genome browser (http://genome.ucsc.edu/cgi-bin/hgGateway?org=Cat&db=felCat4). The contigs were used to aid in the design of further probes for rescreening of the BAC library to isolate clones encompassing *CMAH* and flanking regions (clones RE14 and 182P23; sequences included in GenBank accession code KJ027519). Final, contiguous, complete sequences of selected BAC clones covering the *CMAH* were achieved using SMRT sequencing. Briefly, BAC clone DNA was sent to the Yale Center for Genome Analysis. SMRTbell libraries were prepared as previously described according to the manufacturer’s instructions (PacBio, CA, USA). Sequencing was carried out on the PacBio RS II (PacBio) using standard protocols for long-insert libraries and *de novo* assembled using hierarchical genome assembly process software (PacBio).

### PCR analysis of the *CMAH-*deletion region in mammalian species

PCR primers CMAH_FOR and CMAH_REV were designed to span the entire deleted region of ferret *CMAH* (see accession code JX036482 for primer sequences). These primers amplify a 7-kb PCR product from ferret genomic DNA (Zyagen). Primer walking and Sanger sequencing using BigDye v3.1 and BigDye v1.1 and deaza dGTP (Applied Biosystems) were used in combination to determine the sequence of this repeat-rich 7-kb region of the ferret genome (the sequence has been deposited in the GenBank database with accession code JX036482). The same sequencing process was used to sequence the fragment from a second, independent ferret sample. DNA from this second ferret sample was extracted (DNeasy Blood and Tissue Kit, Qiagen) from ferret kidney tissue provided by the Institute of Medical and Veterinary Science. Animal tissue for various members of the Mustelidae family (Supplementary Table 3) was obtained from the Burke Museum of Natural History and Culture, University of Washington. Genomic DNA from the tissues were isolated as per the manufacturer’s instructions (DNeasy Blood and Tissue Kit, Qiagen). DNA obtained was quantified using a nanodrop (NANODROP 2000, Thermo Scientific), and 10 ng of DNA was used in PCR reactions. CMAH exon PCR products were amplified using Go-Taq polymerase, purified (Qiagen PCR Purification Kit, 28106) and sequenced for verification as before. DNA samples were run on a 3% agarose gel stained with 5% (v/v) ethidium bromide (Sigma).

*CMAH* exon primers were designed for the most highly conserved exons in the *CMAH* gene (Supplementary Table 2). Such regions were determined through the alignment of the coding domain sequence of the CMAH messenger RNA for several eukaryotes such as: cat (accession codes EF127684.1 and NM_001244985.1), human (FJ794466.1), mouse (NM_007717.5, NM_001111110.2, NM_001284519.1, NM_001284520.1), rat (NM_001024273.1), maccaca monkey (NM_001032856.1), chimpanzee (NM_001009041.1) and wild pig (NM_001113015.1). Messenger RNA sequences were aligned using the ClustalW alignment tool in MacVector (ver. 11.0.2, MacVector Inc.). Highly conserved regions were identified in exon 3, exon 5, exon 8, exon 11 and exon 12 of the *CMAH* gene. Primers were designed to be at least 18 nucleotides long, with a minimum annealing temperature of 50 °C, and allowed to contain only a single-nucleotide change per organism. All primers were ordered and made by Sigma (see Supplementary Table 2 for primer sequences). PCR products generated were sequenced to confirm the intended exons were amplified from each animal sample. Genomic DNA of cat (GC-130F), dog (GD-150F) and human (GH-180F) used as PCR controls were purchased from Zyagen.

### Propagation and purification of IAV

Adherent Madin Darby canine kidney (MDCK) cells were obtained from the WHO Collaborating Centre for Reference and Research on Influenza (VIDRL, Melbourne). Cells were grown in Eagle’s minimum essential medium supplemented with 1% penicillin/streptomycin, 1% GlutaMAX and 10% fetal calf serum at 37 °C. Human influenza strains A/Perth/16/2009 (H3N2) and A/California/04/2009 (pH1N1), also obtained from the VIDRL, were passed in MDCK cells in Eagle’s minimum essential medium (1% GlutaMAX) containing low levels of TPCK (N-tosyl-L-phenylalanyl chloromethyl ketone) treated trypsin (1 μg ml^−1^) to facilitate infection of cells. The viral supernatant was harvested after 48 h and concentrated by polyethylene glycol precipation overnight at 4 °C. Standard sucrose density centrifugation was implemented to purify the resuspended polyethylene glycol-precipitated virus according to routine procedures^68^. The purified virus was inactivated by 20-min exposure to ultraviolet light and subsequently buffer exchanged to 20 mM deuterated phosphate buffer pH 7.1 and 70 mM NaCl.

### MUN NA inhibition assay

Inhibition of IAV NA was quantitatively assessed using the fluorescent substrate 4-methylumbelliferyl *N*-acetyl-α-D-neuraminic acid (MUN, Sigma-Aldrich)^69^,^70^,^71^. The 10-μl reaction mixture containing 0.1 mM MUN, 1 μM OC and 1 μl of the purified ultraviolet-inactivated virus in reaction buffer (50 mM sodium acetate, 6 mM CaCl_2_, pH 5.5) was prepared in a black 96-well plate on ice. Different dilutions of virus in triplicate were analysed to identify the highest virus concentration, which is still completely inhibited by 1 μM OC. The reaction was incubated at 37 °C with 900 r.p.m. shaking for 20 min and then stopped by adding 0.25 M glycine pH 10.

### ^1^H NMR-based NA activity assay

All enzyme reactions were performed at 310 K in 20 mM sodium acetate buffer containing 6 mM CaCl_2_, pH 5.5, the optimal pH for NA activity. In a standard ^1^H NMR experiment, a spectrum of each individual reaction mixture containing 1 mM of one of the four sialyllactose substrates (3′SL^Ac^, 3′SL^Gc^, 6′SL^Ac^ and 6′SL^Gc^) was acquired at *t*=0 min. After addition of purified ultraviolet-inactivated virus (A/California/04/2009 pH1N1, A/Perth/16/2009 H3N2) ^1^H NMR spectra were recorded every 30 s over a total time of 20 min with 8 numbers of scans. Substrate conversions could be calculated based on the decrease of the absolute peak intensities of the sialyllactose H3eq-signals. Field variations, differences in baseline correction and background noise have been taken into consideration by applying an error of ±7.5% to the obtained rates.

Similarly, the NA activity of pH1N1 and H3N2 purified virus samples was measured with a substrate mixture of 1 mM 3′SL^Ac^, 3′SL^Gc^, 6′SL^Ac^ and 6′SL^Gc^ in the absence and presence of 50 μM OC at 310 K. The virus concentration was identical as in STD NMR experiments to ensure a complete blocking of the NA’s active site under STD NMR conditions.

### STD NMR experiments

All STD NMR spectra were acquired in Shigemi Tubes (Shigemi) with a Bruker 600 MHz Avance spectrometer at 283 K using ^1^H/^13^C/^15^N gradient cryoprobe equipped with z-gradients and a STD NMR set-up similar to previous experiments using whole-rotavirus particles^48^,^49^. The virus was saturated on resonance at −1.0 p.p.m. and off-resonance at 300 p.p.m. with a cascade of 60 selective Gaussian-shaped pulses of 50-ms duration. A 100-μs delay between each pulse was applied, resulting in a total saturation time of 3 s. A relaxation delay of 4 s was used. A total of 1,024 scans per STD NMR experiment were acquired and a WATERGATE sequence was used to suppress the residual HDO signal. Spin-lock filtre with 5-kHz strength and duration of 10 ms was applied to suppress protein background. Substrate concentrations of 2 mM for 3′SL^Ac^, 3′SL^Gc^, 6′SL^Ac^ and 6′SL^Gc^ were used in all STD NMR set-ups in the presence of 50 μM OC. OC was preincubated with the virus for 10 min at room temperature before adding the various substrates. Control STD NMR experiments were performed with an identical experimental set-up of virus with 50 μM OC, but in the absence of the sialyllactose ligands, to exclude any potential STD NMR signals derived from OC binding to NA. For a second control experiment the virus sample was incubated at 70 °C for 20 min prior to the STD NMR experiment to identify any unspecific binding of the sialyllactose substrates to the virus particle.

### Data mining of publically available glycan array experiments using IAV

Immunofluorescence values were extracted from CFG array experiments (http://www.functionalglycomics.org/, accessed 15 October 2012) in which common sialyllactose or sialyllactosamine structures and common linkages were present that were terminated with either Neu5Ac or Neu5Gc. These structures include 3′SL (Siaα2-3Galβ1-4Glcβ-Sp0), 3′SLN (Siaα2-3Galβ1-4GlcNAcβ-Sp0) and 6′SLN (Siaα2-6Galβ1-4GlcNAcβ-Sp0), where Sia can be either Neu5Ac or Neu5Gc. Data compared are from whole virus and HA. No data were available for NA.

## Author contributions

P.S.K.N. and H.W. conducted analysis of SA expression. P.S.K.N. and M.P.J. designed and conducted molecular and phylogenetic studies in the investigation of the ferret *CMAH* mutation in ferret and animal samples, tested and contributed to writing these sections of the manuscript. R.B., T.H. and M.v.I. designed and conducted NMR analysis of HA and NA specificity and contributed to writing these sections of the manuscript. L.E.H.-T. designed, conducted and wrote the section on data mining. J.A.S., S.W.L., P.L.H. and P.S.K.N. contributed to sequence analysis. M.v.I., S.M.G., A.E.O.T., J.C.P., P.J.C., A.W.P., P.S.K.N. and M.P.J. contributed to design of the study. J.C.P., P.J.C., A.W.P. and M.P.J. conceived the study. M.P.J., P.S.K.N., R.B. and T.H. wrote the manuscript.

## Additional information

**How to cite this article**: Ng, P. S. K. *et al.* Ferrets exclusively synthesize Neu5Ac and express naturally humanized influenza A virus receptors. *Nat. Commun.* 5:5750 doi: 10.1038/ncomms6750 (2014).

**Accession codes**: Nucleotide sequence data from this manuscript have been deposited in the GenBank database with the following accession codes: ferret *CMAH*-deleted region (JX036482), BAC clone CH237-182P23 (KJ027518) and BAC clone CH237-446P7 (KJ027519).

## Supplementary Material

Supplementary Information Supplementary Figures 1-9, Supplementary Tables 1-3, and Supplementary References
