# Supplementary material for: Ferrets exclusively synthesize Neu5Ac and express naturally humanized influenza A virus receptors
Source: Nat Commun. 2014 Dec 17;5:5750. doi: 10.1038/ncomms6750 (PMC4351649; doi:10.1038/ncomms6750)
Supplement: Supplementary Information — Supplementary Figures 1-9, Supplementary Tables 1-3, and Supplementary References [file ncomms6750-s1.pdf]

SUPPLEMENTARY INFORMATION NCOMMS-14-03934C

Supplementary Figure 1

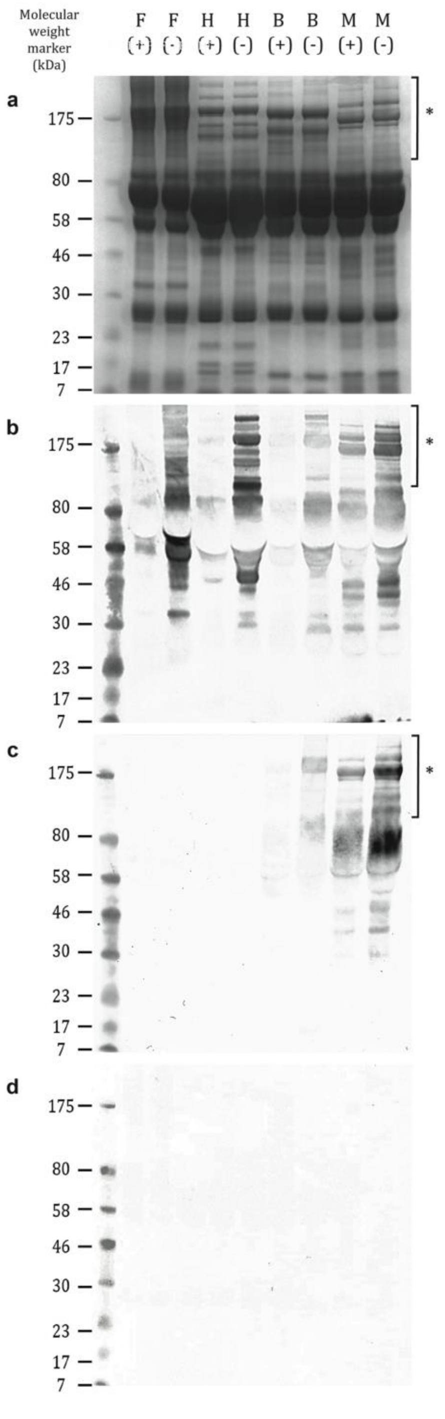

Supplementary Figure 1 | Western blot results showing the absence and presence of Neu5Gc in several selected eukaryotic organisms. (*) represents blot region shown in Figure 2. (a) Coomassie staining results. **(b)** Western blot tested with lectin SNA. **(c)** Western blot tested with anti-Neu5Gc antibody (Gc-Free). **(d)** Western blot tested with Neu5Gc control antibody (Gc-Free).

Supplementary Figure 2a

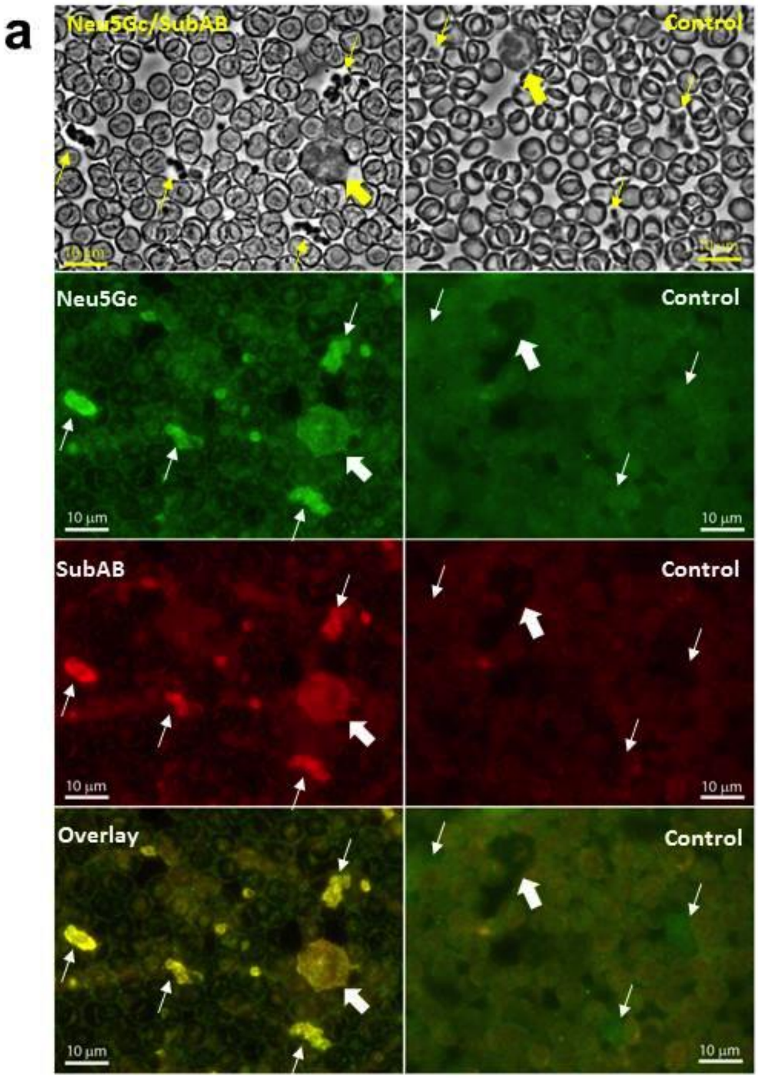

Supplementary Figure 2b

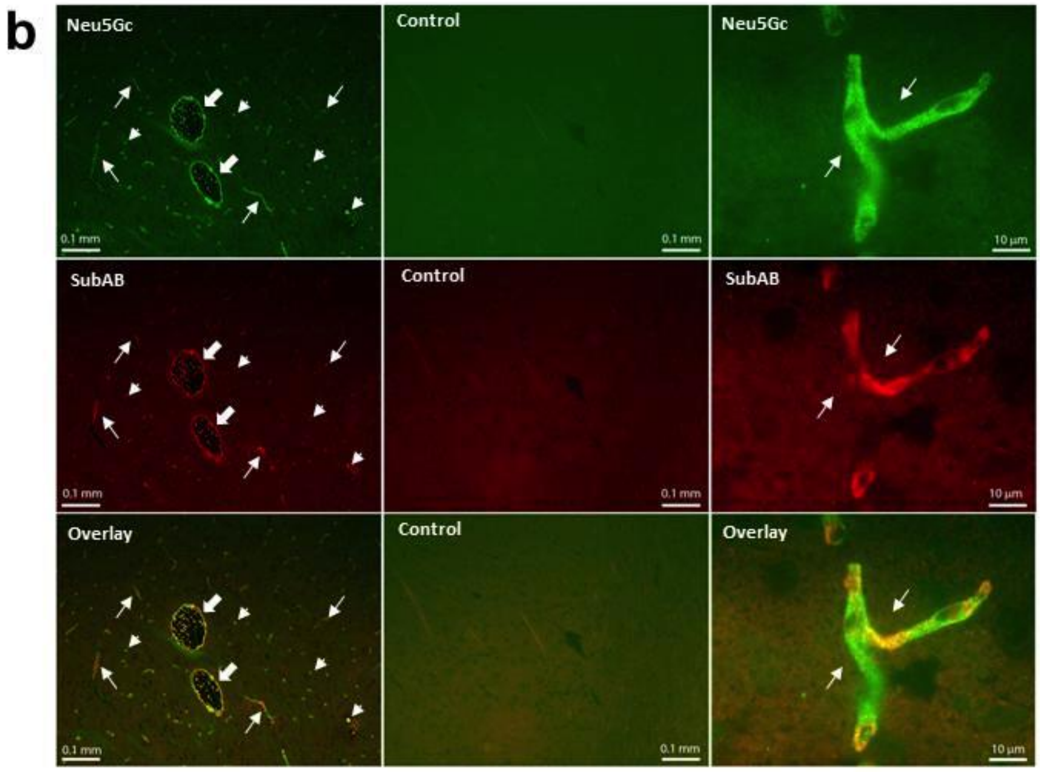

Supplementary Figure 2c

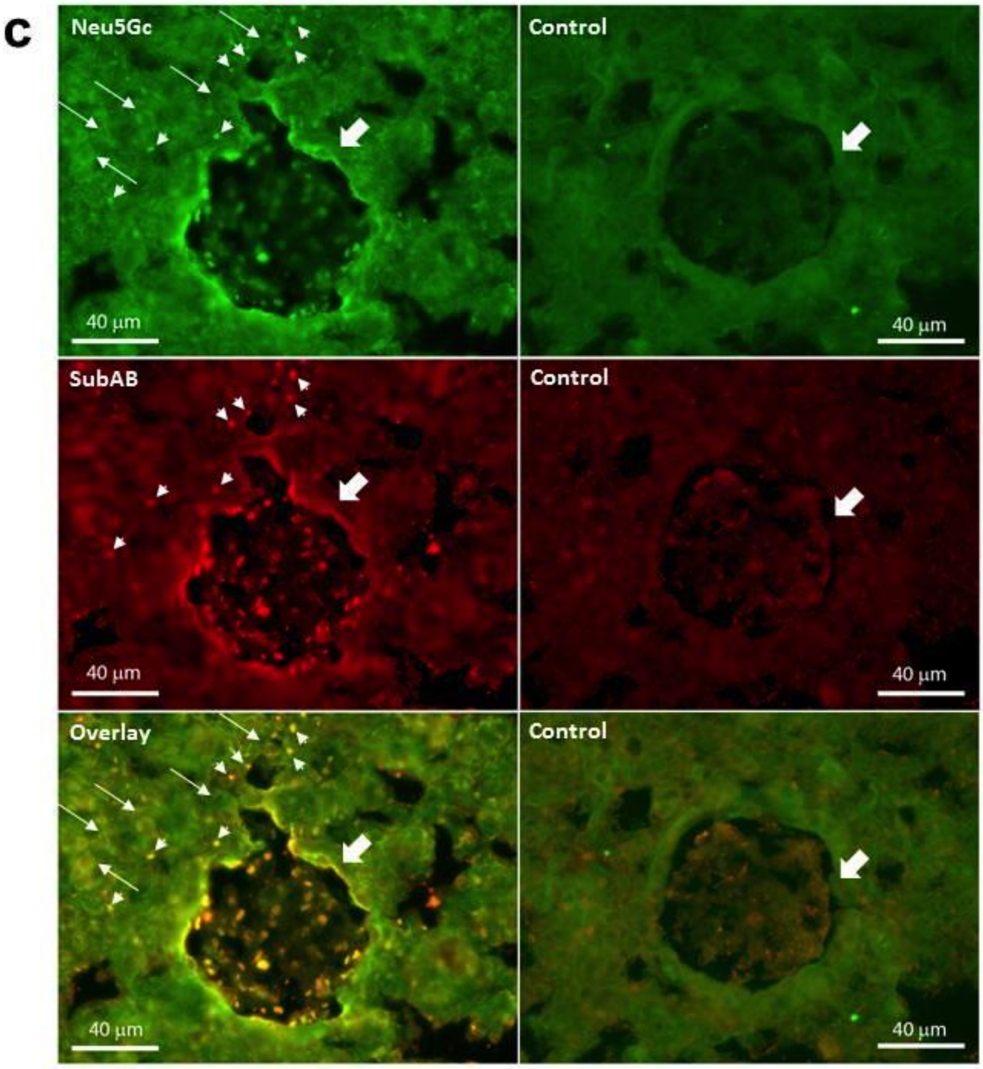

Supplementary Figure 2d

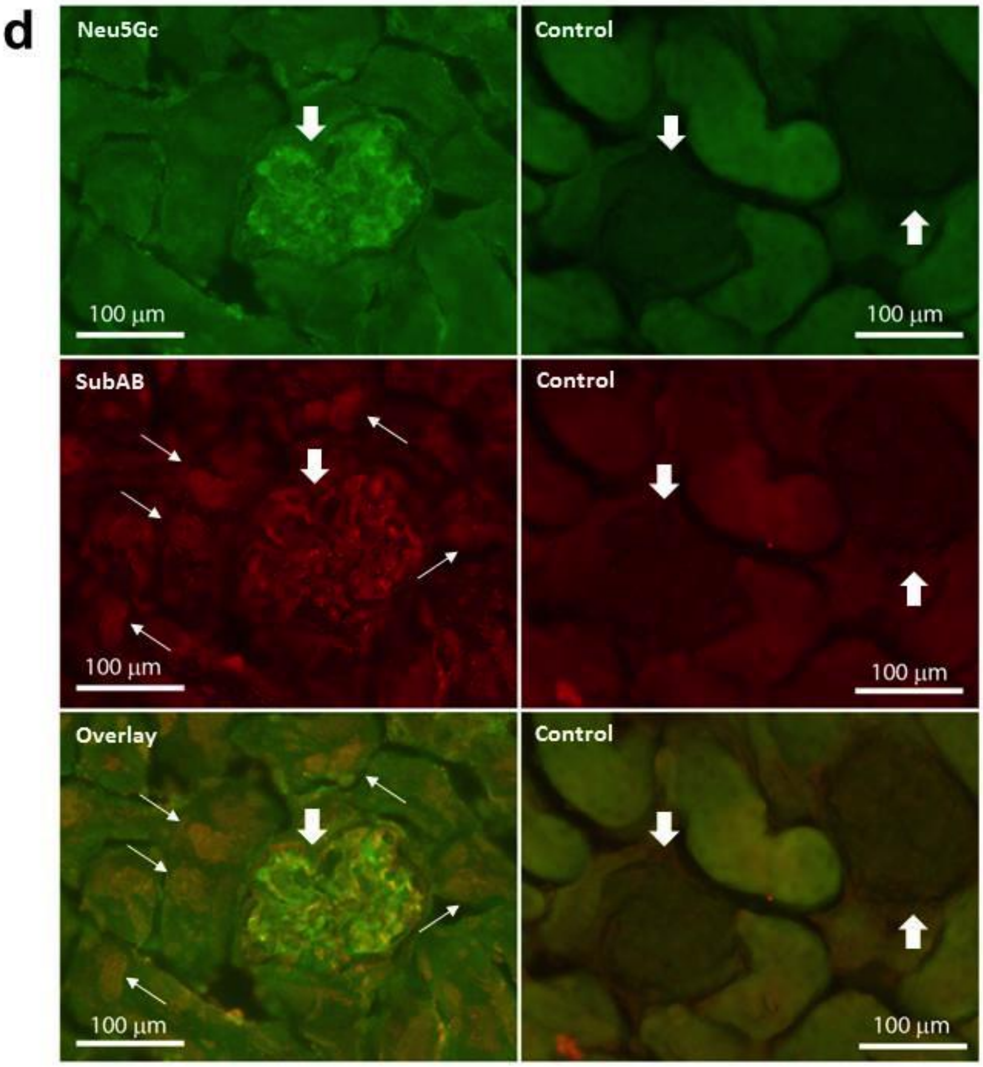

Supplementary Figure 2e

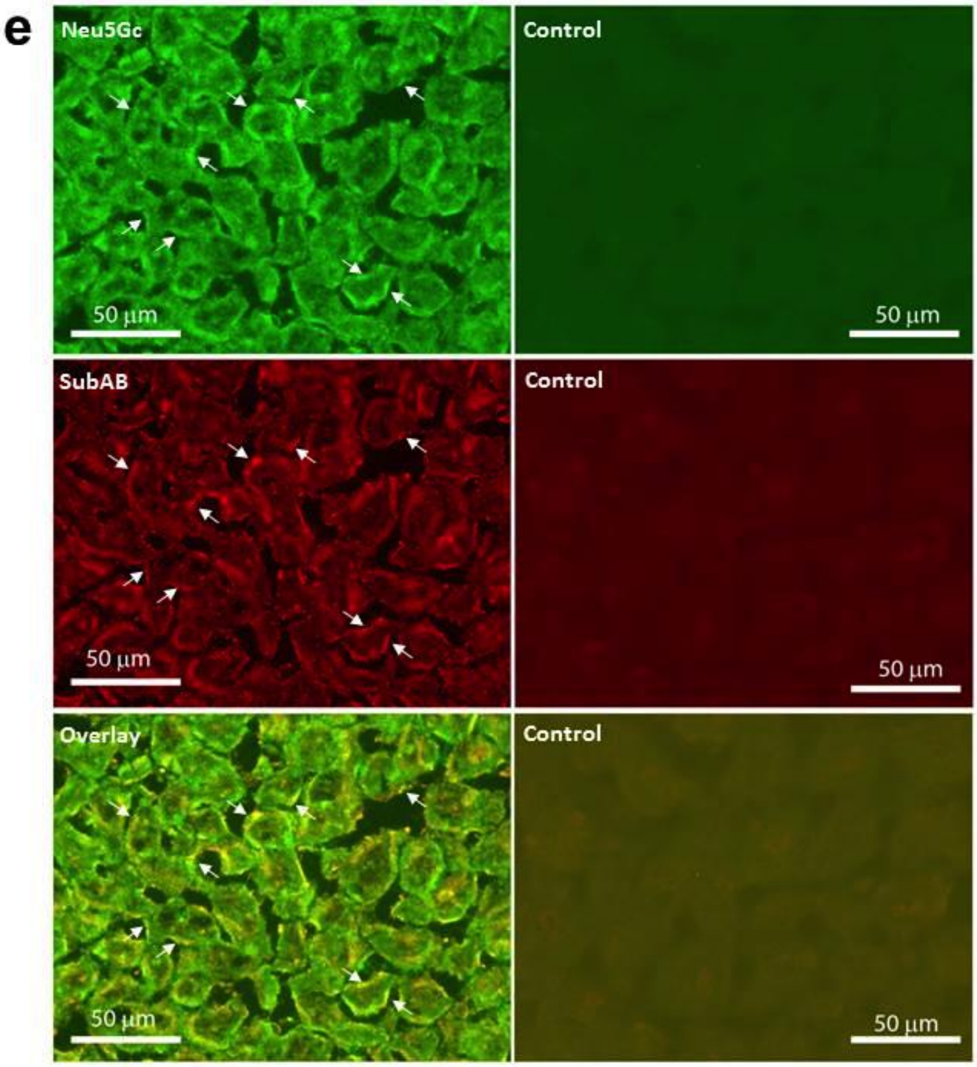

Supplementary Figure 2f

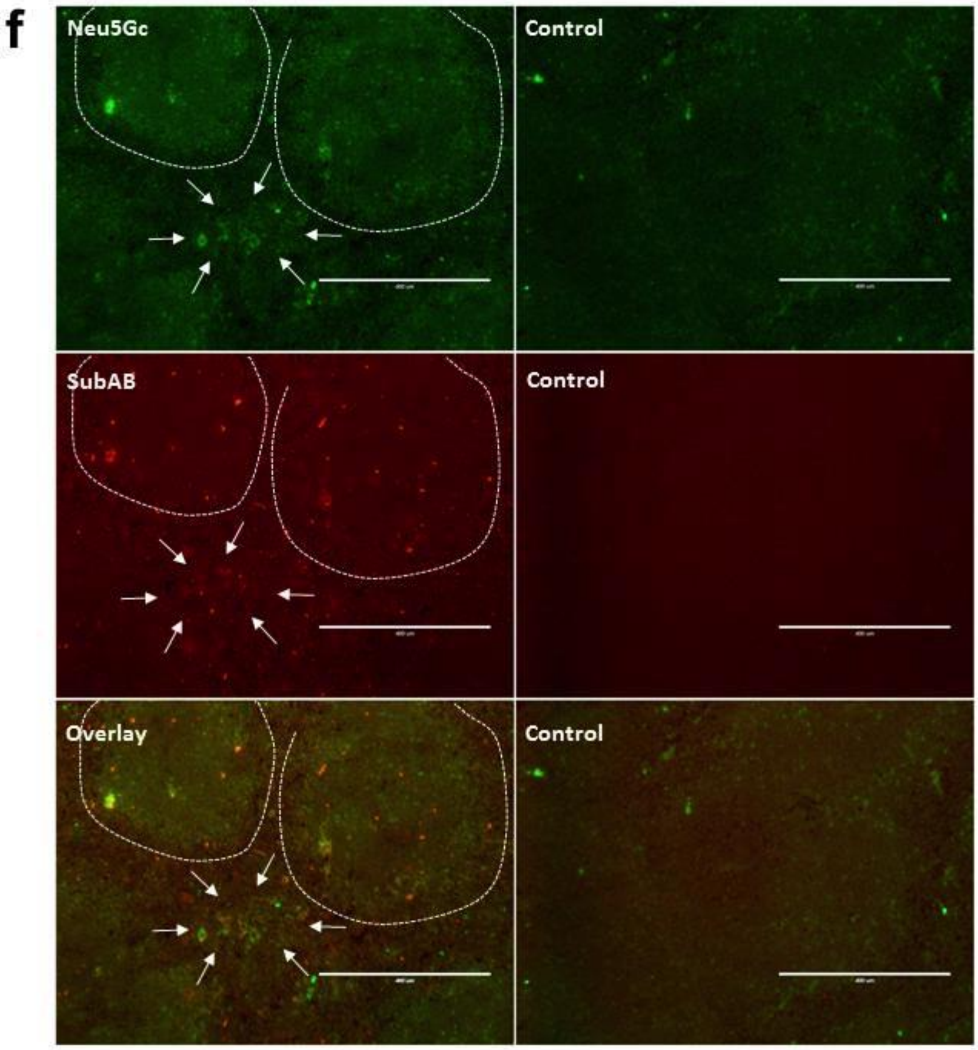

Scale bars, 400 µm.

Supplementary Figure 2g

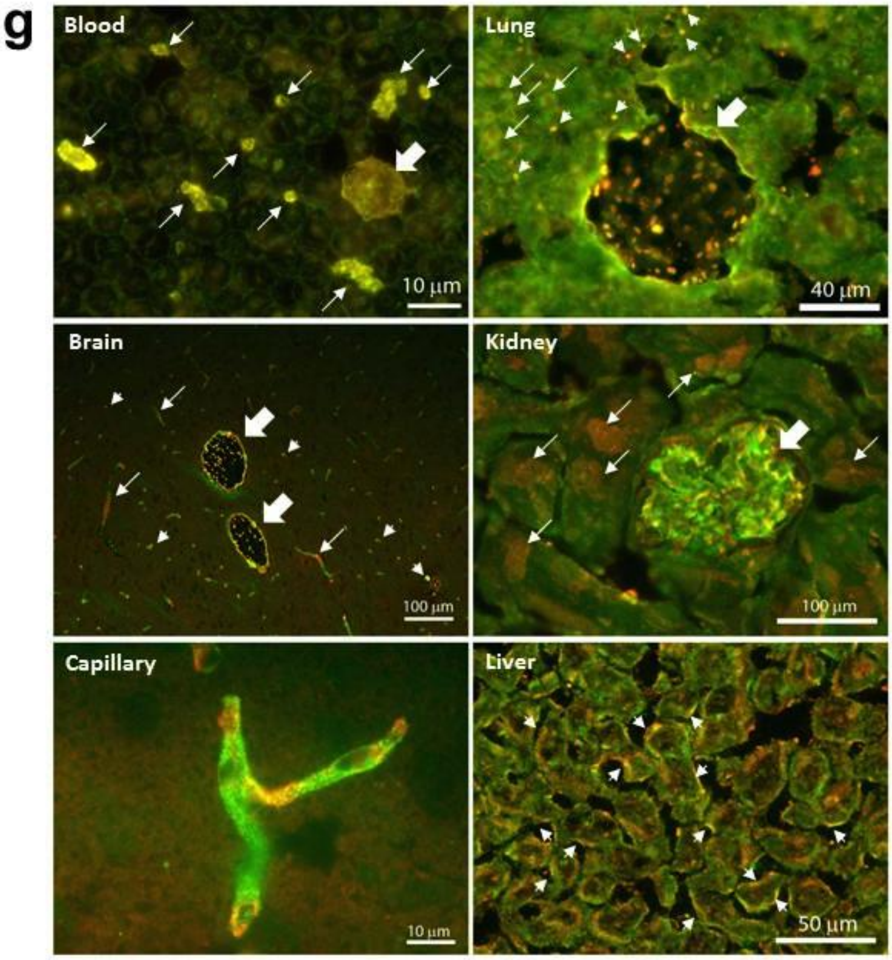

Supplementary Figure 2h

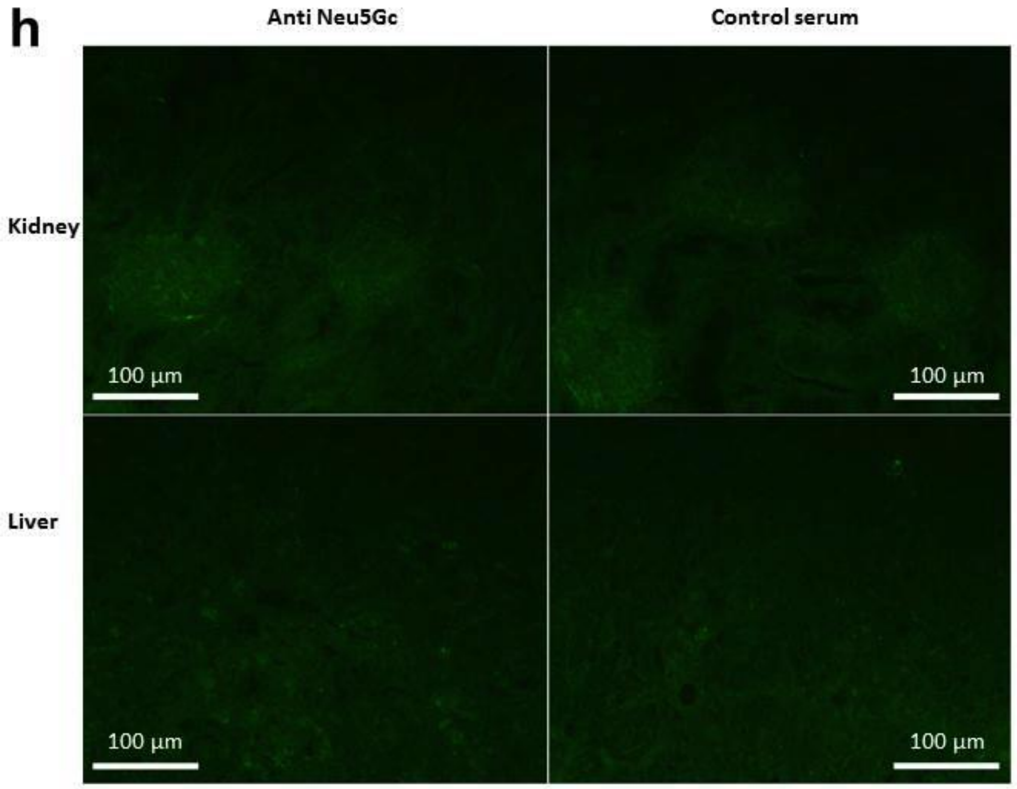

Supplementary Figure 2i

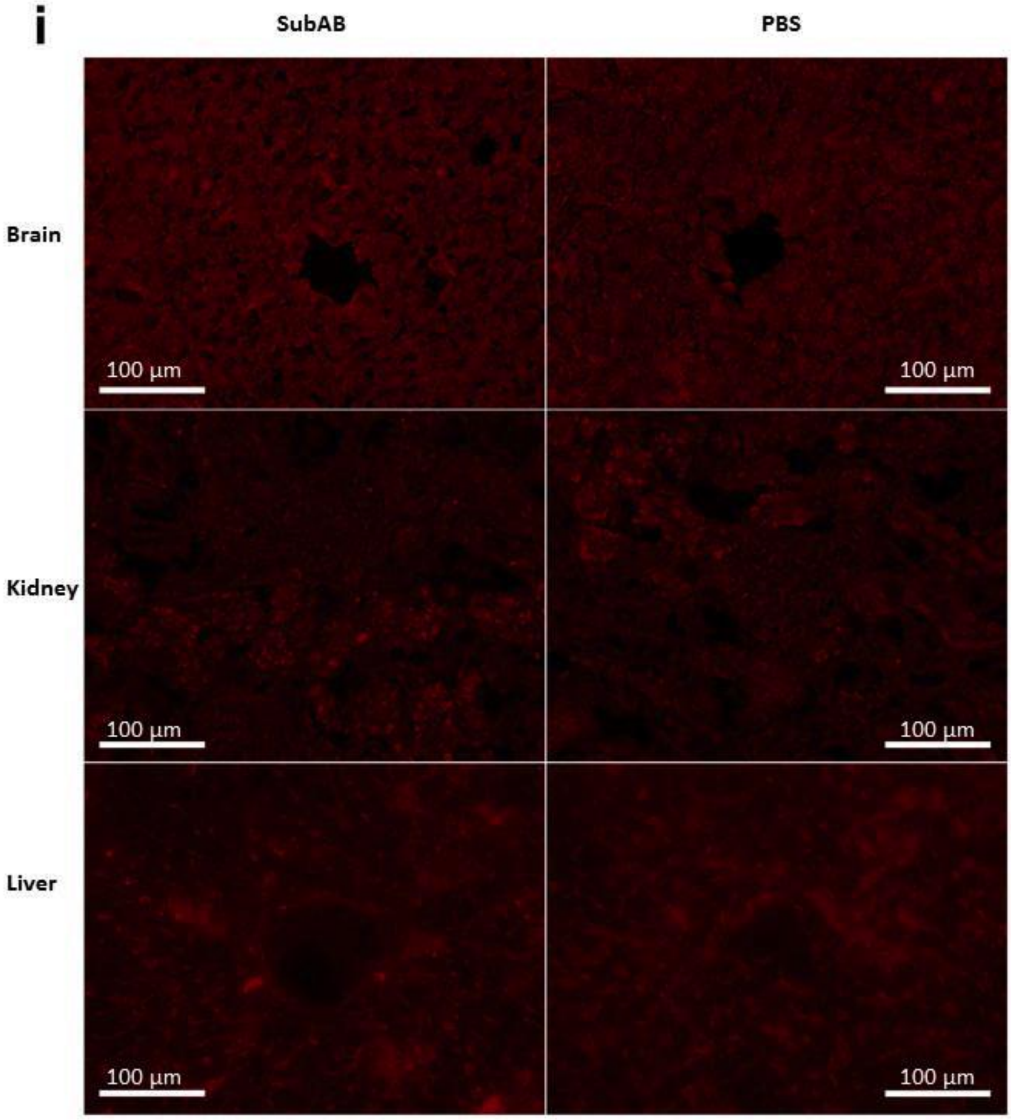

Supplementary Figure 2 | Immunofluorescence images showing SubAB and anti-Neu5Gc binding sites, respectively, detected in mouse and ferret tissues as labelled. (a) mouse blood, bold arrows: polymorph nuclear cells; arrows: platelets. **(b)** mouse brain, bold arrows: small blood vessels; arrows/arrowheads: capillaries (sagittal/transverse planes). **(c)** mouse lung, bold arrows: small blood vessels; arrows: alveolar epithelial cells; arrowheads: capillaries. **(d)** mouse kidney, bold arrows: glomeruli; arrows: renal tubular epithelial cells. **(e)** mouse liver, arrows: liver sinusoidal endothelial cells. **(f)** mouse spleen, dotted line circles: spleen white pulps; arrows: a red pulp area; scale bars: 400 µm. **(g)** mouse organ summary. **(h)** ferret tissue Neu5Gc. **(i)** ferret tissue SubAB.

Supplementary Figure 3a

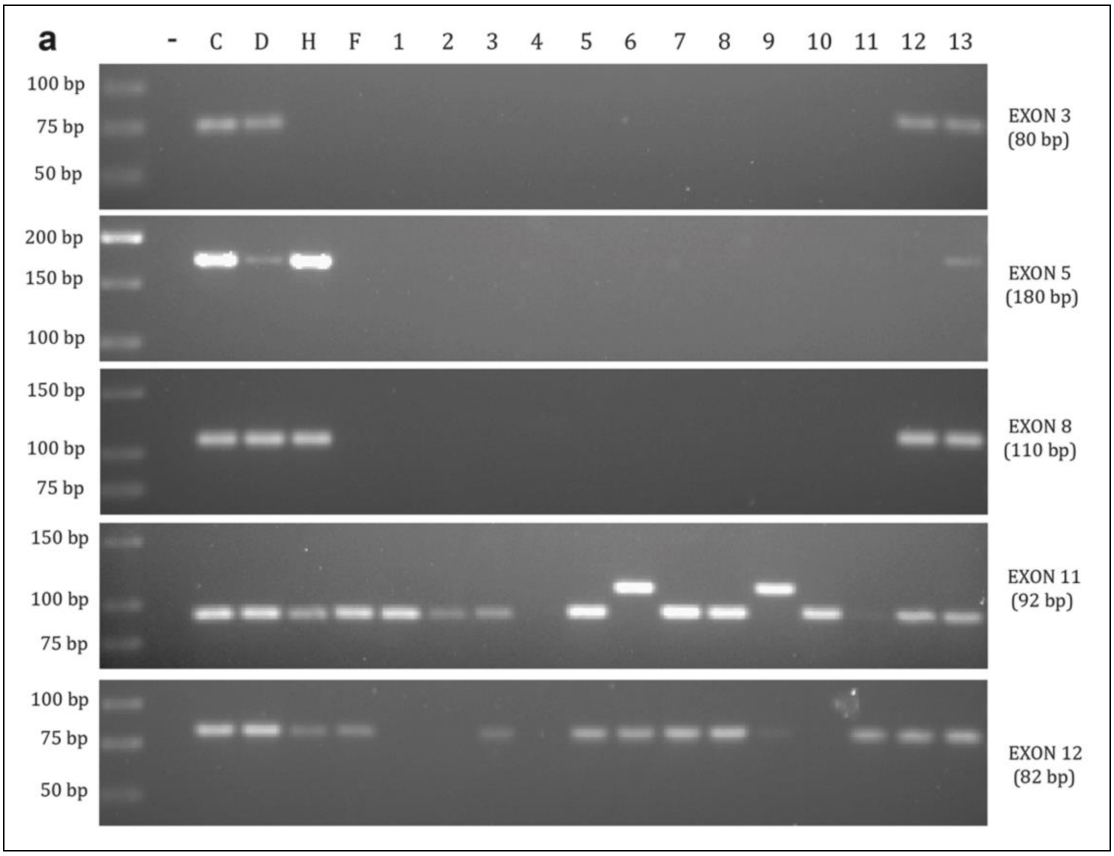

Supplementary Figure 3 | CMAH exon PCR analysis for various members of the carnivora. (a) CMAH exon PCR reactions of exon 3 (80 bp), exon 5 (180 bp), exon 8 (110 bp), exon 11 (92 bp) and exon 12 (82 bp) for cat (C), dog (D), human (H) and ferret (F) and a range of eukaryotes ranging from several different subfamilies in the Mustelidae family respectively. Refer #1 - #11 as listed in Supplementary Table 3; #1 – *Gulo gulo* (Wolverine), #2 – *Martes americana* (American marten) #3 – *Martes pennant* (Fisher), #4 – Mephitis mephitis (Striped skunk), #5 – *Mustela ermine* (Stoat), #6 – *Mustela frenata* (Long tailed weasel), #7 – *Mustela nivalis* (Least weasel), #8 – *Mustela putorius* (European polecat), #9 – *Mustela vison* (American mink), #10 – *Procyon lotor* (Raccoon), #11 – *Taxidea taxus* (American badger). Samples #12 – *Urocyon cinereoargenteus* (Gray fox) and #13 – *Vulpes vulpes* (Red fox) are samples from the Canidae family.

Supplementary Figure 3b

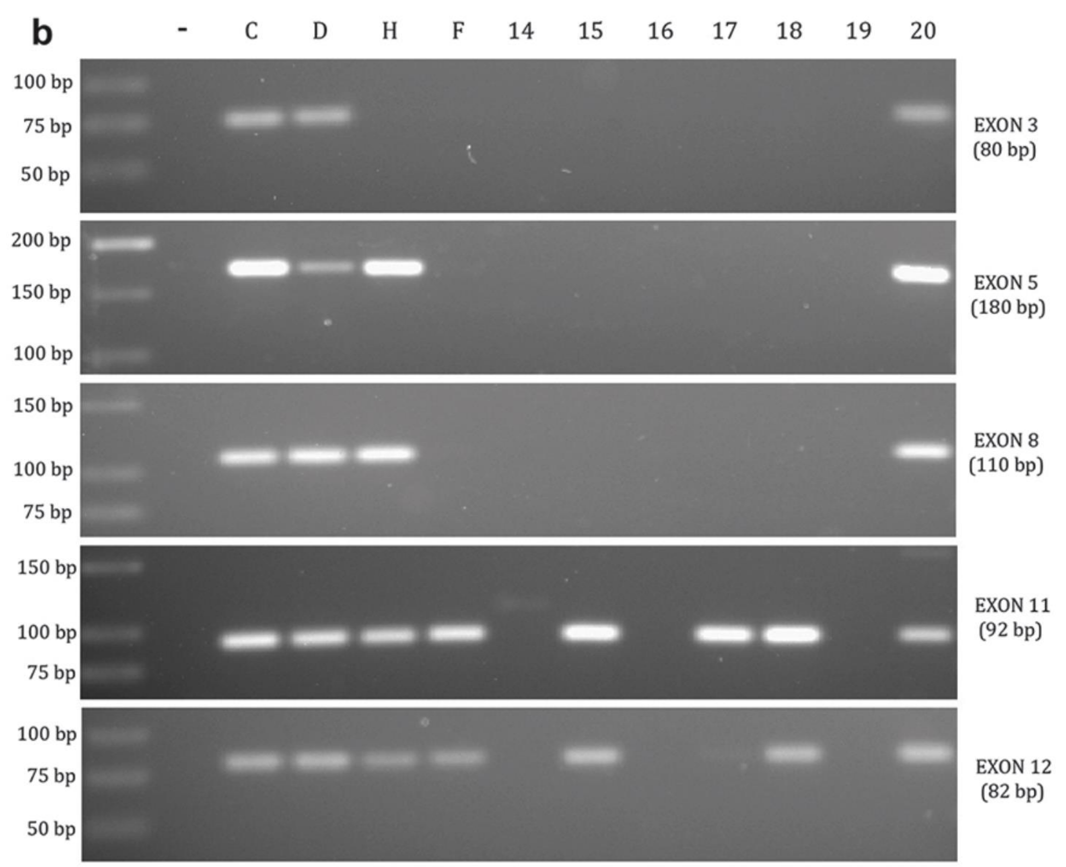

Supplementary Figure 3 (b) *CMAH* exon PCR reactions of exon 3 (80 bp), exon 5 (180 bp), exon 8 (110 bp), exon 11 (92 bp) and exon 12 (82 bp) for cat (C), dog (D), human (H) and ferret (F) conducted on extra eukaryotes from the Mustelidae family and including other families of the Caniformia taxa. Refer #14 - #20 as listed in Supplementary Table 3; #14 – *Ailurus fulgens* (Red panda), #15 – *Enhydra lutis* (Sea otter), #16 – *Eumetopias jubatus* (Stellar sea lion), #17 – *Lontra canadensis* (North American sea otter), #18 – *Mustela sibirica* (Siberian weasel), #19 – *Phoca vitulina* (Harbor seal), #20 – *Ursus americanus* (American black bear).

Supplementary Figure 3c

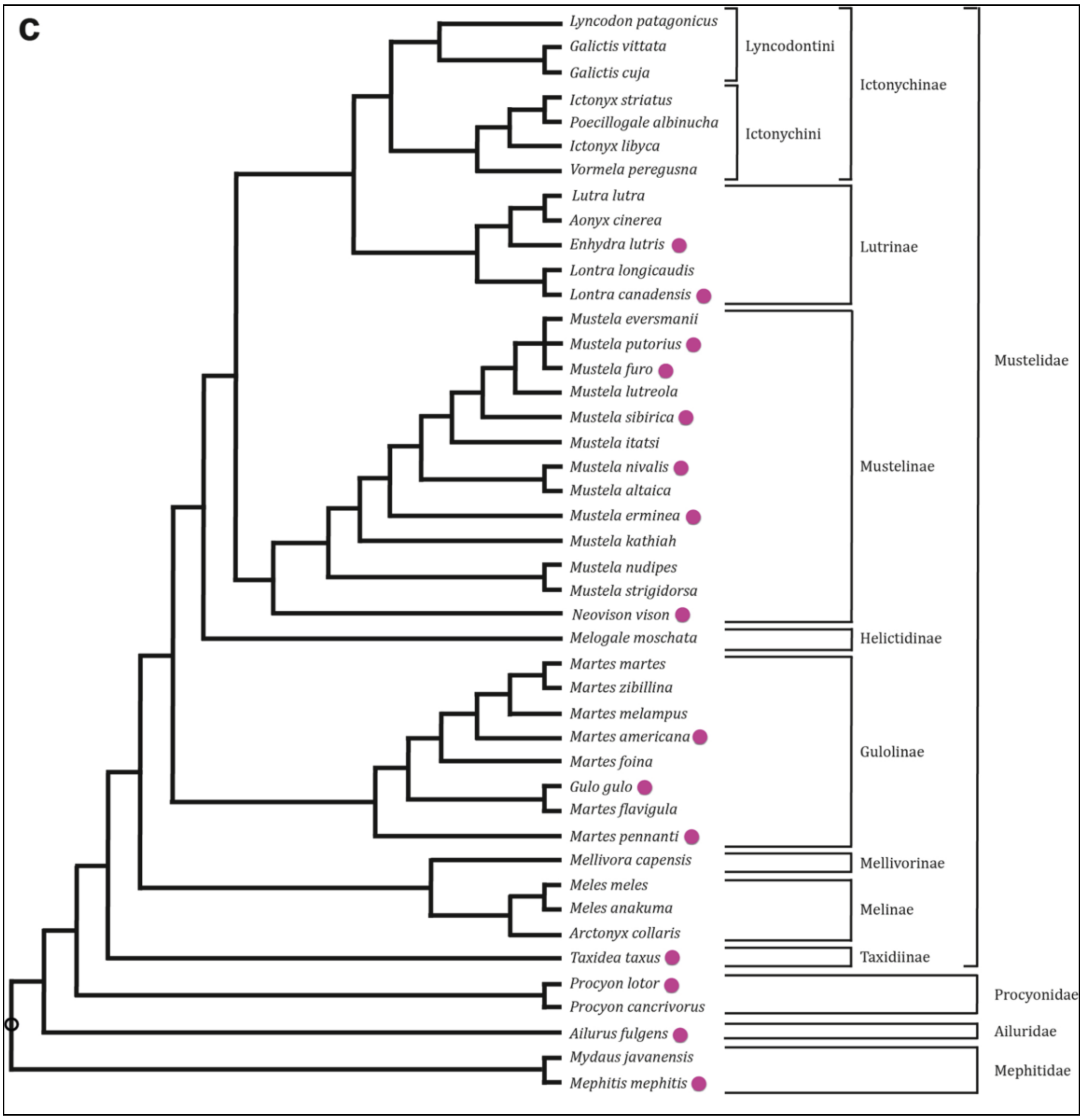

Supplementary Figure 3 (c) Detailed schematic diagram highlighting the phylogeny relationships of the Musteloidea with its respective lineages; Mustelidae, Procyonidae, Ailuridae and Mephitidae. Samples marked with a dot denote samples tested in this study. The magenta dot indicates the *CMAH* gene found to have the same apparent deletion as ferret *CMAH*. Note that *Mustela frenata* (JEB1645) is not represented in the diagram and belongs to the Mustelinae subfamily of Mustelidae. Diagram is modified from Sato *et al.* 2012[72].

Supplementary Figure 3d

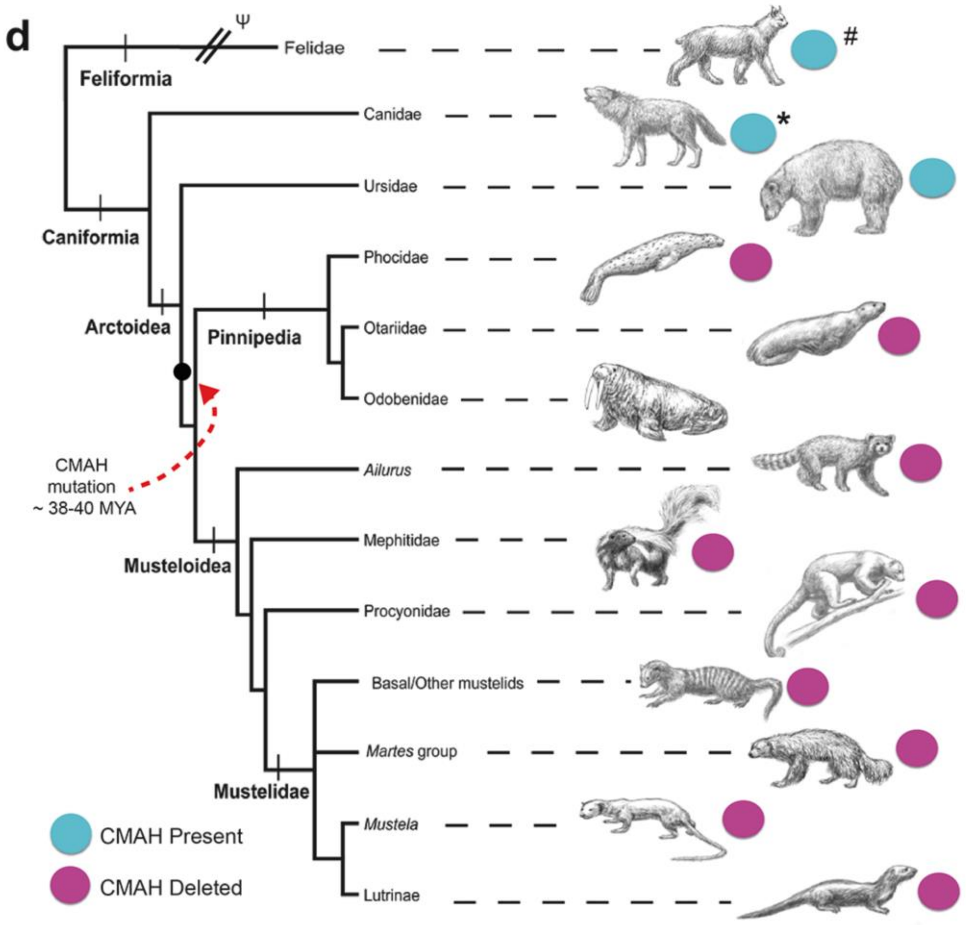

Supplementary Figure 3 (d) Schematic diagram highlighting the evolutionary relationships of the Carnivora order (mammals). Samples used in this study were derived from the Canidae family (*Urocyon cinereoargenteus* and *Vulpes vulpes*) and the three lineages of the Arctoidea line comprising of Ursidae family (*Ursus americanus*), Pinnipedia family (*Eumetopias jubatus* of subfamily Phocidae, *Phoca vitulina* of subfamily Otariidae) and the Musteloidea family (Refer to Supplementary Figure 3c). Samples marked with a dot denote samples tested in this study. The magenta dot indicates the *CMAH* gene found to have the same apparent deletion as ferret *CMAH*. Samples marked with a cyan dot are samples which amplified all *CMAH* exons tested, and do not contain the extensive 9 exon deletion found in ferret. A red arrowed line indicates a black dot that shows the origin of the 9 exon *CMAH* deletion estimated to have occurred 38-40 MYA when the Arctoidea family split in to the Ursidea, Pinnipedia and Musteloidea lineages. Symbol (#) denotes genus *Felis catus*, domestic cat sample used as a PCR control representing the Felidae family of Felimormia family. Presence of Neu5Gc can denote blood typing group in domestic cats[31,32]. Symbol (*) denotes that denotes genus *Canis lupus familiaris*, domestic dog sample used as PCR control representing the Canidae family. *CMAH* can differentiate between different species of dogs from different continents[28,29]. Symbol (Ψ) denotes that only the Felidae genus of the Feliformia branch of Carnivora was tested. Diagram is modified from Flynn *et al.* 2005[73].

Supplementary Figure 4

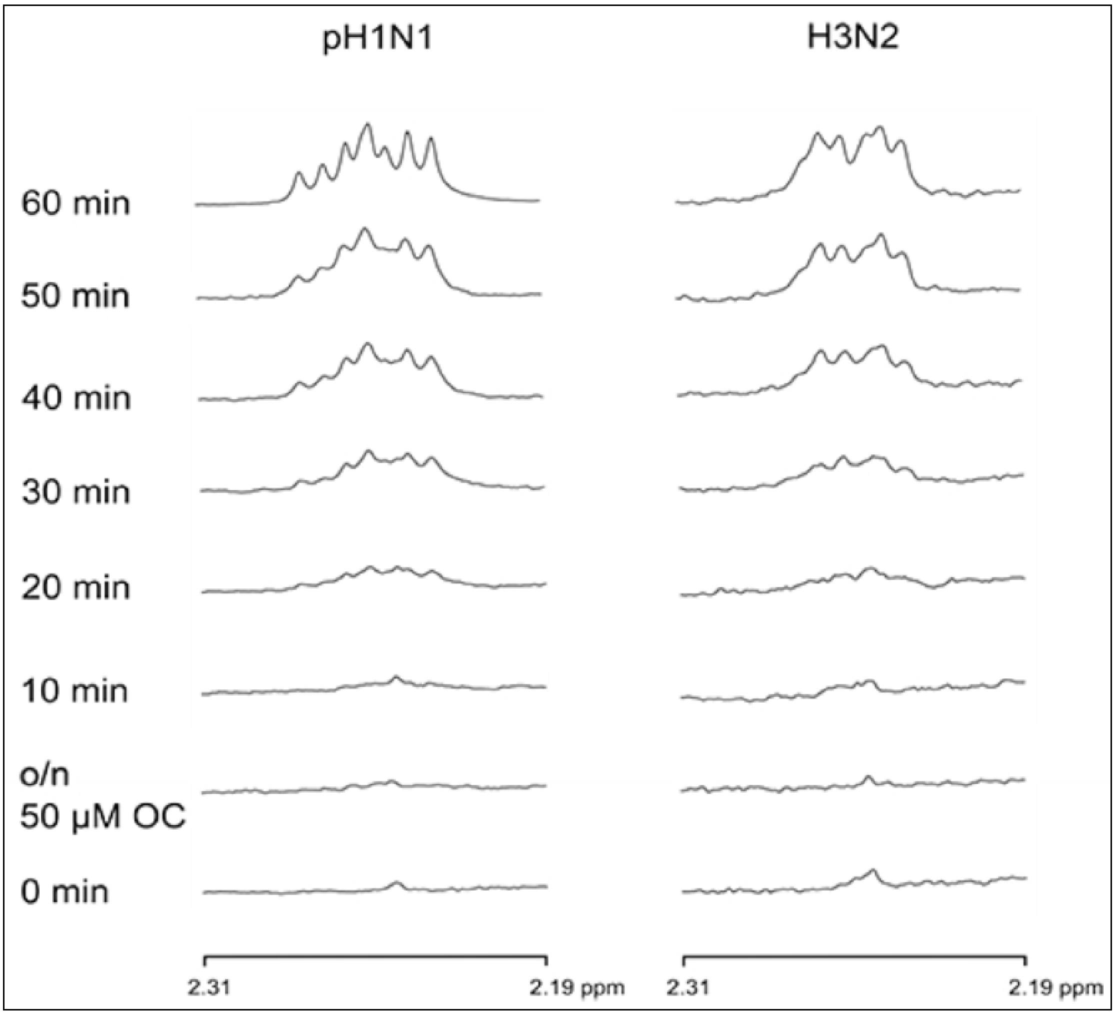

Supplementary Figure 4 | Neuraminidase activity assay. ^{1}H NMR based neuraminidase activity assay using pH1N1 (A/California/04/2009) and H3N2 (A/Perth/16/2009) virus with an equimolar mixture of 1 mM substrates (3´SL^{Ac}, 3´SL^{Gc}, 6´SL^{Ac}, 6´SL^{Gc}). An identical virus concentration was used as described for the STD NMR experiments. The ^{1}H NMR spectra depict the region of the equatorial H3 protons of β-Neu5Ac and β-Neu5Gc that are released by the neuraminidase cleavage reaction. Spectra were recorded without virus (0 min) and after addition of virus for one hour with consecutive acquisition of ^{1}H NMR spectra every ten minutes. An increase in ^{1}H NMR signal intensities indicates that the neuraminidase is active and thereby affirming that the virus remained intact after purification and UV-inactivation. Under identical conditions in the presence of 50µM oseltamivir carboxylate no product can be detected after over night incubation at 37 °C demonstrating that the active site of NA is completely blocked.

Supplementary Figure 5

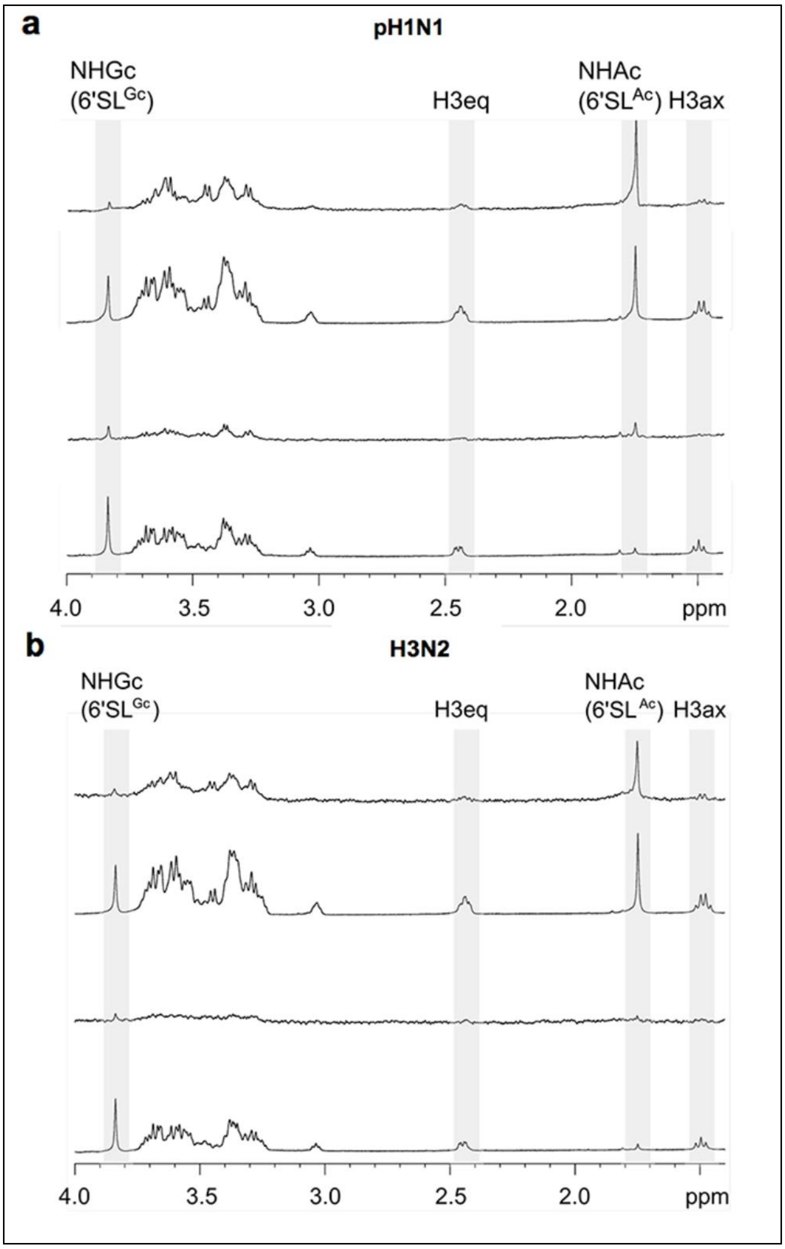

Supplementary Figure 5 | Whole virus STD NMR. ^{1}H NMR (bottom panel) and STD NMR (top panel) spectra of 2 mM 6´SL^{Gc} (Neu5Gcα2,6Galβ1,4Glc) with influenza A viruses **(a)** pH1N1 (A/California/04/2009) and **(b)** H3N2 (A/Perth/16/2009), respectively. The grey boxes highlight the axial and equatorial H3 protons (H3ax, H3eq), the *N*-acetamido methyl (NHAc) and the methylene (NHGc) protons that are clearly distinguishable between 6´SL^{Ac} and 6´SL^{Gc}. Very low STD NMR signal intensities indicate weak affinity of pH1N1 and H3N2 influenza A viruses towards 6´SL^{Gc}. To obtain an equimolar ligand mixture, 2 mM 6´SL^{Ac} (Neu5Acα2,6Galβ1,4Glc) were added to the sample and another STD NMR (top) and ^{1}H NMR (below) spectra were acquired confirming strong interactions of 6´SL^{Ac} with both pH1N1 and H3N2 viruses as seen in Supplementary Figure 4.

Supplementary Figure 6

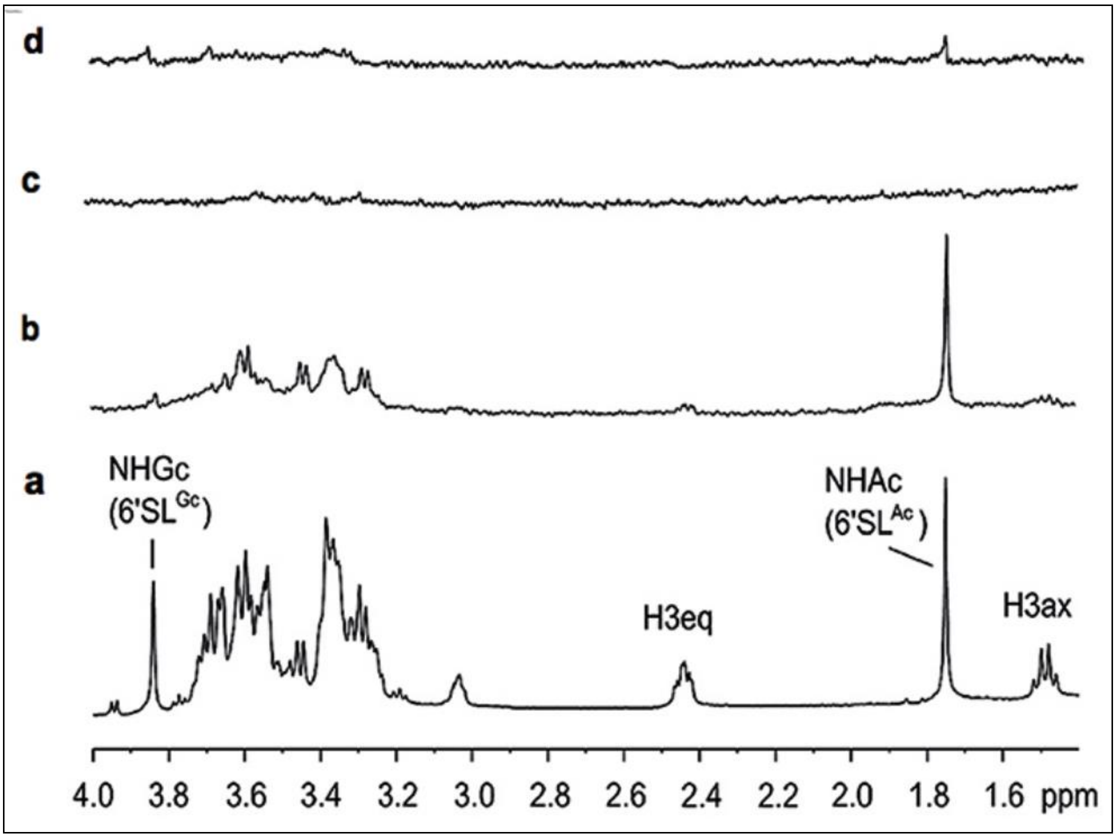

Supplementary Figure 6 | Whole virus STD NMR. **(a)** ^{1}H NMR and **(b)** STD NMR spectra of an equimolar mixture of 2 mM 6´SL^{Ac} (Neu5Acα2,6Galβ1,4Glc) and 6´SL^{Gc} (Neu5Gcα2,6Galβ1,4Glc) with pH1N1 virus (A/California/04/2009) in the presence of 50 µM oseltamivir carboxylate (OC). Shown are the axial and equatorial H3 protons (H3ax, H3eq) and the *N*-acetamido methyl (NHAc) and methylene glycolyl (NHGc) protons of the sialic acid moiety that are clearly distinguishable between 6´SL^{Ac} and 6´SL^{Gc}. A control STD NMR spectrum of 50 µM OC in complex with pH1N1 shows no prominent STD NMR signals **(c)** With low OC concentration, the lower copy number of neuraminidase compared to haemagglutinin on the viral surface shows a very high affinity of OC to neuraminidase provide very poor STD NMR conditions which explains why no STD NMR signals can be detected. At the same time, the active site of neuraminidase is effectively blocked to avoid substrate cleavage. In a second control experiment the virus sample was heat-treated for 20 minutes at 70 °C and a STD NMR spectrum was obtained under otherwise identical NMR experimental conditions as in panel (b) (**d)** Very weak STD NMR signals indicate the specificity of glycan receptor binding to the haemagglutinin of the H1N1 virus.

Supplementary Figure 7

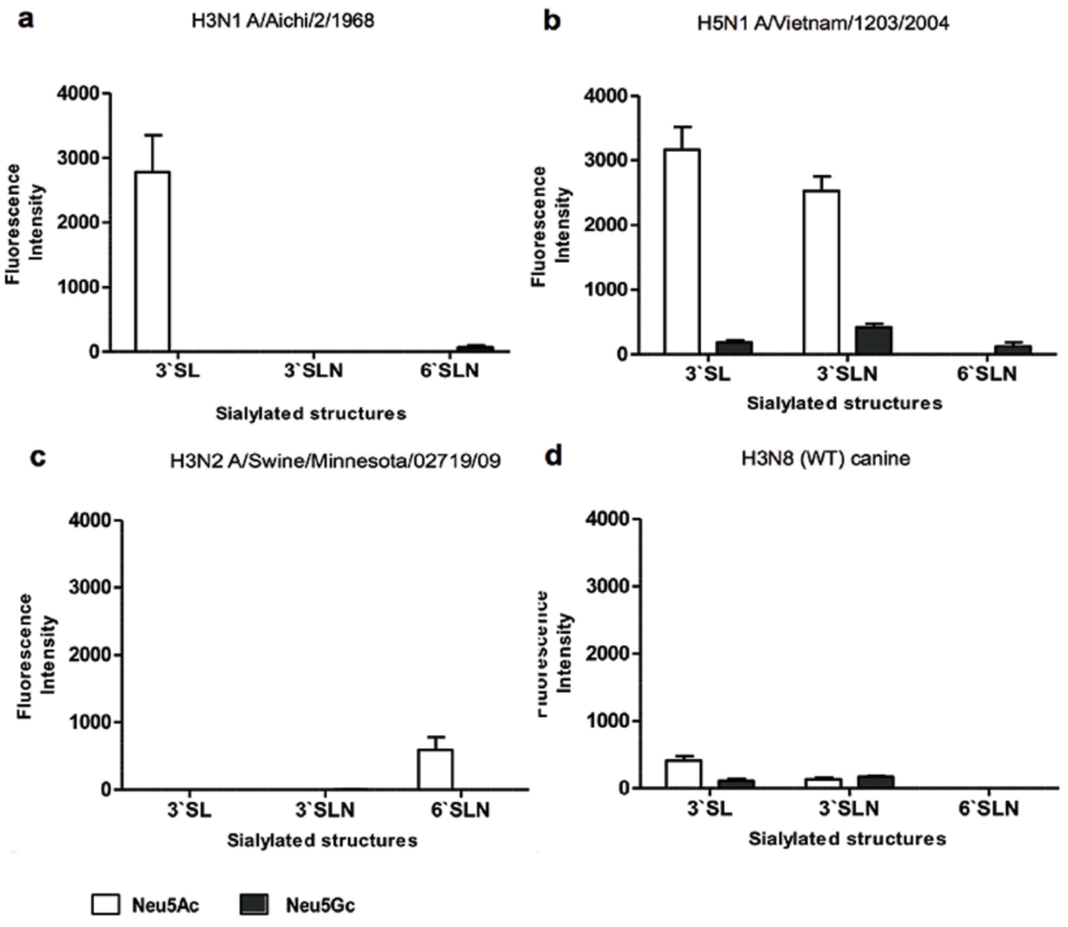

	H3N1		H3N2		H3N8		H5N1	
	Neu5Ac	Neu5Gc	Neu5Ac	Neu5Gc	Neu5Ac	Neu5Gc	Neu5Ac	Neu5Gc
3`SL	2779 ± 572	NB	NB	NB	411 ± 68	110 ± 33	3166 ± 353	185 ± 38
3`SLN	NB	NB	NB	6 ± 4	130 ± 30	172 ± 14	2529 ± 222	417 ± 62
6`SLN	NB	64 ± 26	593 ± 189	NB	NB	NB	NB	125 ± 62

Supplementary Figure 7 | Average fluorescent intensities for binding of whole influenza A virus to sialyllactose structures. Data analysed from Consortium for Functional Glycomics website (http://www.functionalglycomics.org/, accessed 15th October 2012) data source showed after each sample type tabulated below for; **(a)** H3N1 A/Aichi/2/1968 (primscreen_2161), **(b)** H5N1 A/Vietnam/1203/2004 (primscreen_4912), **(c)** H3N2 A/Swine/Minnesota/02719/09 (primscreen_3555) and **(d)** H3N8 H3N8 (WT) Canine (primscreen_4155). Raw data values are tabulated. n=4 and the values shown are the mean ± 1 SD.

Supplementary Figure 8

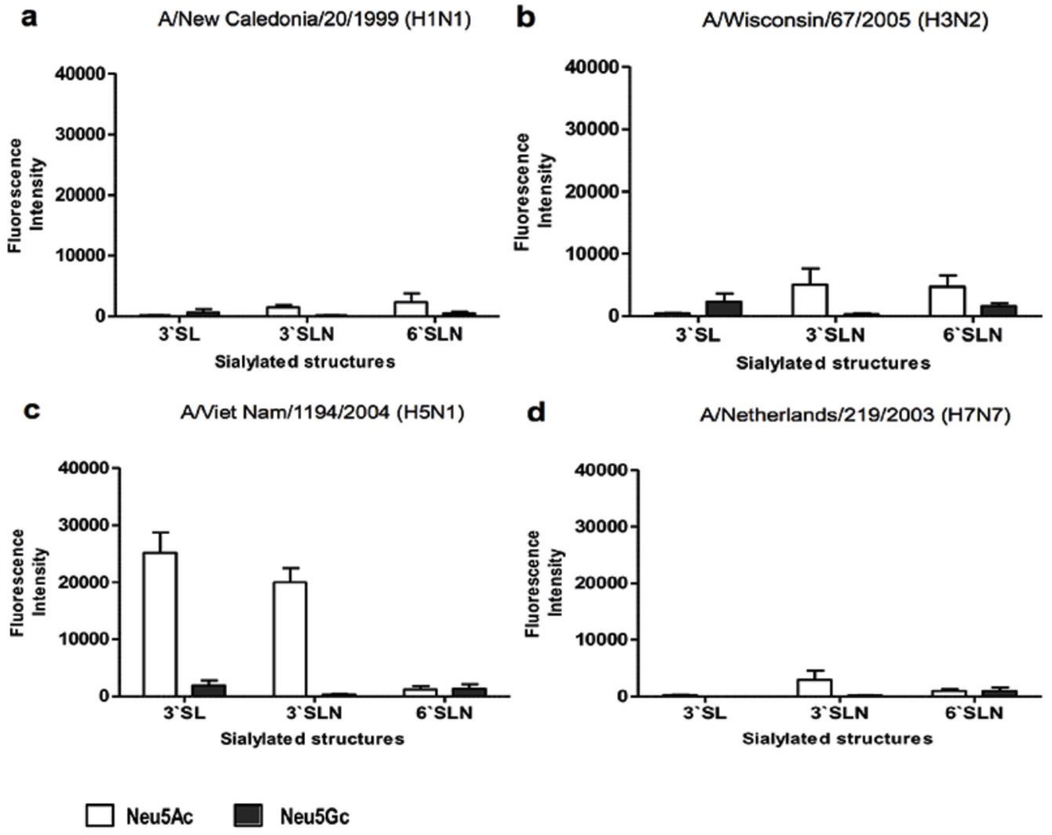

	H1		H3		H5		H7	
	Neu5Ac	Neu5Gc	Neu5Ac	Neu5Gc	Neu5Ac	Neu5Gc	Neu5Ac	Neu5Gc
3`SL	104 ± 40	599 ± 567	380 ± 64	2313 ± 1291	25081 ± 2593	1835 ± 981	230 ± 22	NB
3`SLN	1452 ± 410	120 ± 22	4985 ± 2599	288 ± 80	19973 ± 2448	305 ± 112	2962 ± 1615	180 ± 44
6`SLN	2323 ± 1389	455 ± 258	4673 ± 1868	1595 ± 488	1188 ± 574	1302 ± 787	973 ± 328	999 ± 599

Supplementary Figure 8 | Average fluorescent intensities for binding of recombinant haemagglutinin to sialyllactose structures. Data analysed from Consortium for Functional Glycomics website (http://www.functionalglycomics.org/, accessed 15th October 2012) source showed after each sample type tabulated below for; **(a)** Influenza A H1 hemagglutinin (A/New Caledonia/20/1999 H1N1)(primscreen_3434), **(b)** Influenza A H3 haemagglutinin (A/Wisconsin/67/2005 H3N2)(primscreen_3435), **(c)** Influenza A H5 haemagglutinin (A/Viet Nam/1194/2005 H5N1)(primscreen_3436), and **(d)** Influenza A H7 haemagglutinin (A/Netherlands/219/2003 H7N7)(primscreen_3437). Raw data values are tabulated. n=4 and the values shown are the mean ± 1 SD.

Supplementary Figure 9

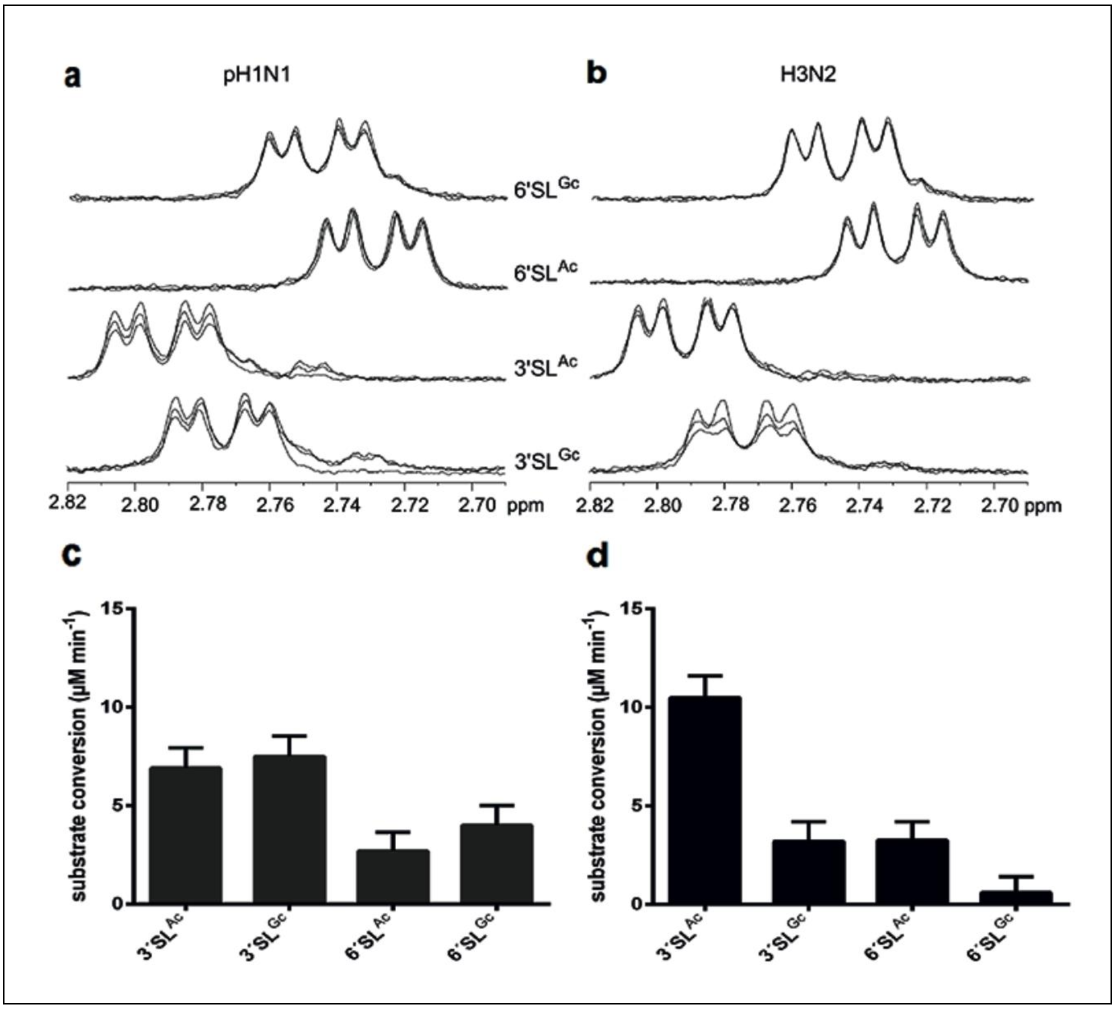

Supplementary Figure 9 | Neuraminidase activity assay. ^{1}H NMR based neuraminidase activity assay to determine substrate conversion rates for four sialosides (6´SL^{Ac}, Neu5Acα2,6Galβ1,4Glc; 6´SL^{Gc}, Neu5Gcα2,6Galβ1,4Glc; 3´SL^{Ac}, Neu5Acα2,3Galβ1,4Glc; 3´SL^{Gc}, Neu5Gcα2,3Galβ1,4Glc) by **(a)** pH1N1 virus (A/California/04/2009, left panel) and **(b)** H3N2 virus (A/Perth/16/2009, right panel), respectively. The decline of the ^{1}H NMR sialoside H3eq proton signals is directly proportional to substrate depletion and product formation of **(c)** pH1N1 and **(d)** H3N2. The conversion rate was therefore calculated based on the signal decline in a successive series of forty ^{1}H NMR spectra over 20 minutes at 37 °C. The figure shows for each reaction the overlay of three ^{1}H NMR spectra (after 0, 10 and 20 minutes).

Supplementary Tables

Supplementary Table 1: Primers used to make probes for BAC library screening

Primer name	Primer sequence (5' - 3')
FAM65B_FOR	AGCCTCTTGAGGATCAG
FAM65B_REV	CAAGATGGCACAAAAG
RE14_FOR	GACCTCGGCATGAATTAGGA
RE14_REV	TTCCCAGGAACAAGCAAATC
182P23_FOR	GGAAGATTTCACGGAGTCCA
182P23_REV	CCCTGTCAAATCATGCACAC
LRRC16_FOR	TCTTCACCAGAGTCGCTAGA
LRRC16_REV	CCCTTTCTGTTTCAGTTTCA

Supplementary Table 2: CMAH Exon PCR Primers

Exon	Primer name	Primer sequence (5'- 3')	PCR Product size (bp)
3	Exon3_FOR	ATGCACAAAGCACAACTGGA	80
	Exon3_REV	CCAGGCAGCTTCTGTCAAGA	
5	Exon5_FOR	GCCTGCATGGACCTCAAG	180
	Exon5_REV	TGCACTCAGACCACCTGAG	
8	Exon8_FOR	TGCACCAGACCCAATGGG	110
	Exon8_REV	TGACTTTCAGTGGTGGAAAATTTAC	
11	Exon11_FOR	GGATTCCTGGGACTTTG	92
	Exon11_REV	ATCCTGGATAAAAGAGTACTT	
12	Exon12_FOR	GAAACAGATGAGGACTTCA	82
	Exon12_REV	TTATCCTTTCCAAAAGAAAG	

Supplementary Table 3: Members of the Mustelidae family used in this study

Sample #	***Genus species***	***Common name***	***UWMB# /Tissue#*** [a]
1	*Gulo gulo*	Wolverine	41022/JR1187
2	*Martes americana*	American marten	81059/EEM701
3	*Martes pennanti*	Fisher	81058/KBA1412
4	*Mephitis mephitis*	Striped skunk	81784/RLP017
5	*Mustela erminea*	Stoat	81735/JMW032
6	*Mustela frenata*	Long tailed weasel	81806/JEB1645
7	*Mustela nivalis*	Least weasel	77364/AVM80
8	*Mustela putorius*	European polecat	81876/TNL255
9	*Mustela vison*	American mink	81002/JEB1389
10	*Procyon lotor*	Raccoon	81971/CMC025
11	*Taxidea taxus*	American badger	77861/JEB1082
12	*Urocyon cinereoargenteus*	Gray fox	77676/JR2284
13	*Vulpes vulpes*	Red fox	80736/JEB1453
14	*Ailurus fulgens*	Red panda	78647/HH009
15	*Enhydra lutis*	Sea otter	81995/EEM871
16	*Eumetopias jubatus*	Stellar sea lion	81964/JEB1748
17	*Lontra canadensis*	North American river otter	81969/TNL202
18	*Mustela sibirica*	Siberian weasel	75336/XGZ283
19	*Phoca vitulina*	Harbor seal	81966/JEB1752
20	*Ursus americanus*	American black bear	80613/JEB1344

[a]UWMB # and Tissue # are sample identifiers from the Burke Museum of Natural History and Culture, Genetic Resources Collection, University of Washington, Seattle, WA, USA.

Supplementary References

72 Sato, J. J. *et al.* Evolutionary and biogeographic history of weasel-like carnivorans (Musteloidea). *Molecular phylogenetics and evolution* **63**, 745-757, doi:10.1016/j.ympev.2012.02.025 (2012).

73 Flynn, J. J., Finarelli, J. A., Zehr, S., Hsu, J. & Nedbal, M. A. Molecular phylogeny of the carnivora (mammalia): assessing the impact of increased sampling on resolving enigmatic relationships. *Systematic biology* **54**, 317-337, doi:10.1080/10635150590923326 (2005).
